# Supplementary material for: Malaria parasites undergo a rapid and extensive metamorphosis after invasion of the host erythrocyte
Source: EMBO Rep. 2025 Apr 4;26(10):2545–73. doi: 10.1038/s44319-025-00435-3 (PMC12116788; doi:10.1038/s44319-025-00435-3)
Supplement: Supplementary file 1 — Appendix [file 44319_2025_435_MOESM1_ESM.pdf]

APPENDIX

Page

2 **APPENDIX FIGURE S1** Phenotypic analyses of parasites lacking PV6.

4 **APPENDIX FIGURE S2** Production of parasites producing a PV6- mNeonGreenfusion.

5 **APPENDIX FIGURE S3** Analysis of iRBCs infected with rapamycin-treated parasites detected using ImageStream analysis.

6 **APPENDIX FIGURE S4** Overview of ImageStream analysis.

7 **APPENDIX FIGURE S5** Parasite populations before and after microsphiltration.

8 **APPENDIX TABLE S1** Statistical analysis of data in Figure 2B.

8 **APPENDIX TABLE S2** Comparison of surface area of uninfected erythrocytes (uRBC) and erythrocytes infected with DMSO-treated PV6 DiCre parasite (iRBC).

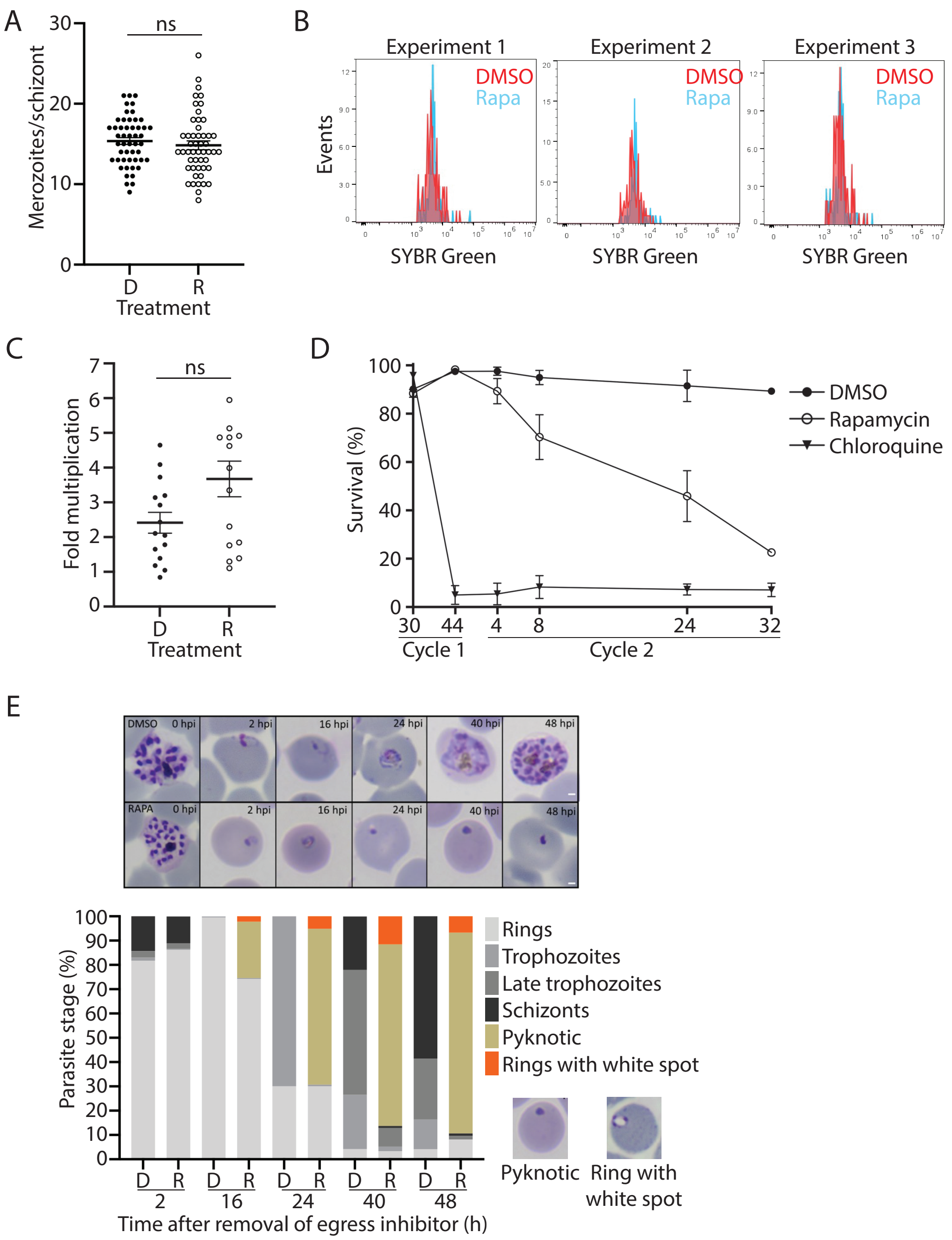

APPENDIX FIGURE S1. Phenotypic analyses of parasites lacking PV6. A. Number of merozoites per schizont in PV6-diCre (PfBLD529) parasites treated with DMSO (D) or rapamycin (R). Results are presented as the average number of merozoites per schizont of three biological replicates (a minimum of 15 schizonts were counted per replicate). Error bars indicate the error of mean. Mann–Whitney U test was performed for statistical analysis (ns: not significant). **B.** DNA content of the schizonts of DMSO-treated and rapamycin-treated PV6-diCre (PfBLD529) parasites. Shown are histograms from three individual experiments representing the DNA content (determined using SYBR Green staining and flow cytometry) of Percoll-purified PV6-diCre (PfBLD529) parasites treated with DMSO (red) or rapamycin (blue). At least 100,000 events were counted per sample (only events representing infected erythrocytes are shown). No obvious difference in the DNA content between the DMSO-treated and

rapamycin-treated parasites was detected. **C.** Invasion rate of DMSO-treated and rapamycin-treated PV6-diCre (PfBLD529) parasites. Schizonts were incubated with fresh erythrocytes and the parasitemia was determined immediately (0 h) and after 24 hours (24 h). The invasion rate was determined as the ratio of the parasitaemia at the two time points (24 h/0 h). Five biological replicates, all in triplicate, were performed. Error bars indicate the error of mean. The Mann–Whitney U test was performed for statistical analysis (ns: not significant). **D.** Survival of PV6-diCre (PfBLD529) parasites treated with DMSO or rapamycin at the trophozoite stage. Parasite viability was determined by staining parasites with Mitotracker DeepRed and SYBR-Green. Invasion in the second cycle was synchronized using ML10. Viable (Mitotracker DeepRed-positive) parasites were compared to the percentage of all SYBR green-positive parasites (dead and viable parasites). Analysis was performed at the indicated time after removal of ML10. Chloroquine was used as control for detection of non-viable parasites. Results are presented as the mean (+/- SD) of two biological replicate assays performed in triplicate. E. Top panel: Giemsa-stained thin films showing the development of DMSO-treated (top row) and rapamycin-treated (bottom row) parasites at the indicated times after removal of the egress inhibitor. The scale bar represents 1 µm. Bottom panel: quantification of parasite developmental stages or phenotype of parasite development at the indicated time after removal of ML10. Rapamycin-treated parasites (R) formed rings but showed an accumulation of rings with white spots and pyknotic forms at an early stage (see examples of these phenotypes under the legend). These parasites did not develop to the trophozoite stage. Results are presented as the means of the count of two biological replicates.

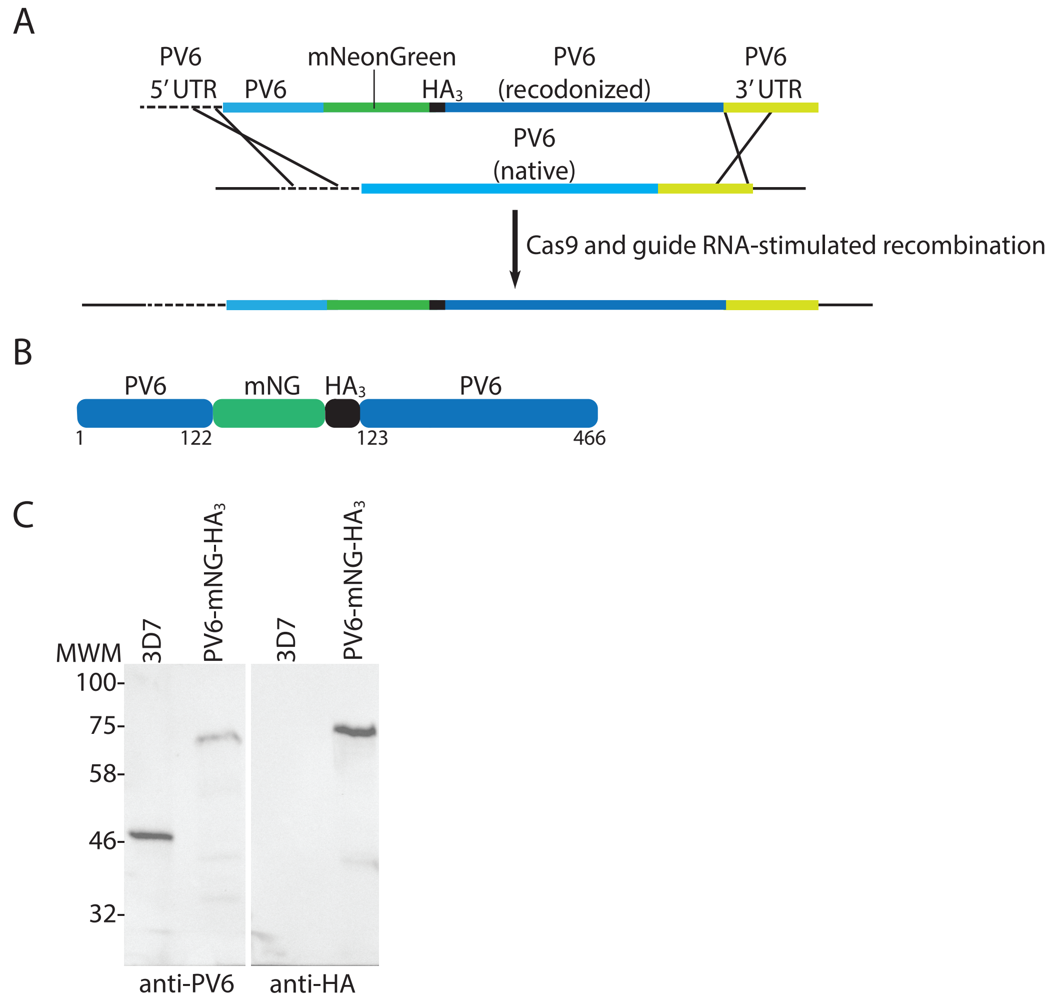

APPENDIX FIGURE S2. Production of parasites producing a PV6-mNeonGreen fusion. A. Outline of the strategy used to produce *Plasmodium falciparum* parasites expressing a mNeonGreen-HA_3 (mNG-HA_3)-tagged version of PV6. This strategy is identical to the strategy used by Fréville *et al.* (Fréville *et al,* 2024) to modify the *pv6* locus. Cas9 and guide RNA-mediated cleavage of the native PV6 coding sequence promotes its replacement with the fusion gene. The coding sequences of mNeonGreen and the HA_3-tag are inserted between codon 122 and 123 in the *pv6* gene. **B.** Cartoon showing the expected protein product after insertion of mNG-HA_3 in PV6 between the AA position 122 and 123. The numbering indicates the amino acid positions in the native PV protein. **C.** Immunoblots on extracts from wild-type (3D7) and transgenic (PV6-mNG-HA_3) parasites using anti-PV6 (left) and anti-HA (right) antibodies. Expected molecular weights: native PV6 – 46.4 kDa (3D7), PV6-mNG – 72.2 kDa (PV6-mNG). Note the absence of a band representing native PV6 in the extract of PV6-mNG parasites, indicating a very high efficiency of integration.

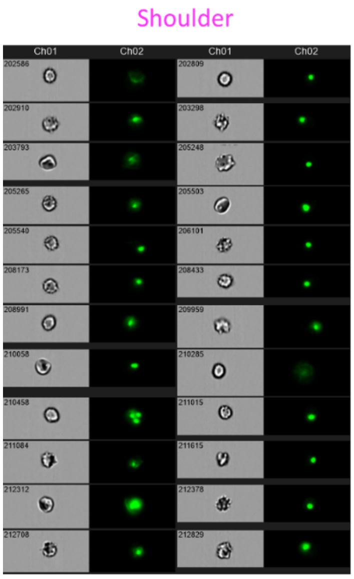

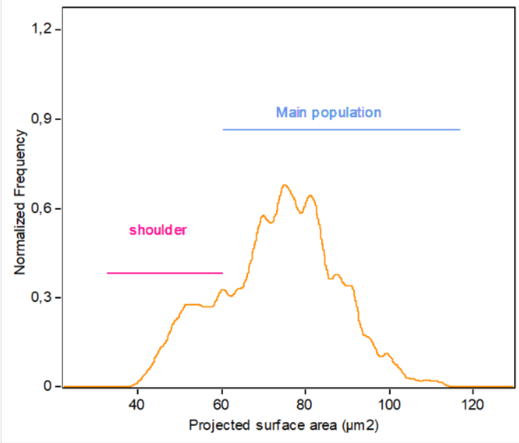

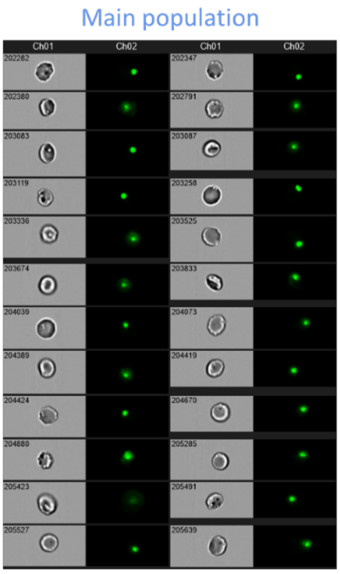

Projected surface area (µm2)

Population	Count	%Gated	Mean	Median
Rings 466 30R KA	1746	100	73,41	74,44
shoulder & Rings 466 30R KA	339	19,4	52,67	53
Main population & Rings 46...	1401	80,2	78,49	77,56

APPENDIX FIGURE S3. Analysis of iRBCs infected with rapamycin-treated parasites detected using ImageStream analysis. The peak representing erythrocytes infected with rapamycin-treated parasite displayed a shoulder, indicating that many small erythrocytes were infected with these parasites. Shown are representative images of parasites in this shoulder (left) and parasites in the main population (right). The shoulder corresponds to small spherocytic and echinocytic (late stages echinocytes i.e echinocytes III and spheroechinocytes) RBCs, while the main population correspond mostly to discocytes and early stages echinocytes (Echinocytes I and II). Shown also are the data, including the surface areas of the populations.

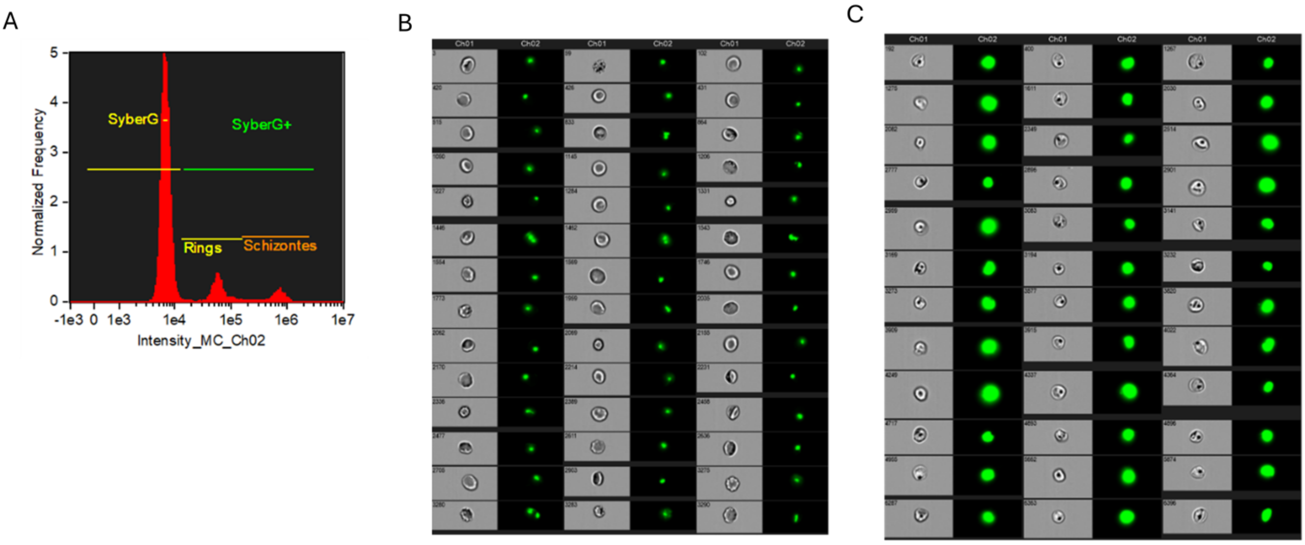

APPENDIX FIGURE S4. Overview of ImageStream analysis. Cultures were stained with SYBR Green and the intensity of the SYBR Green, representing the amount of DNA in the parasites, was determined by flow cytometry (A). This allowed the early-stage parasites (B) to be differentiated from the schizont-stage parasites (C)

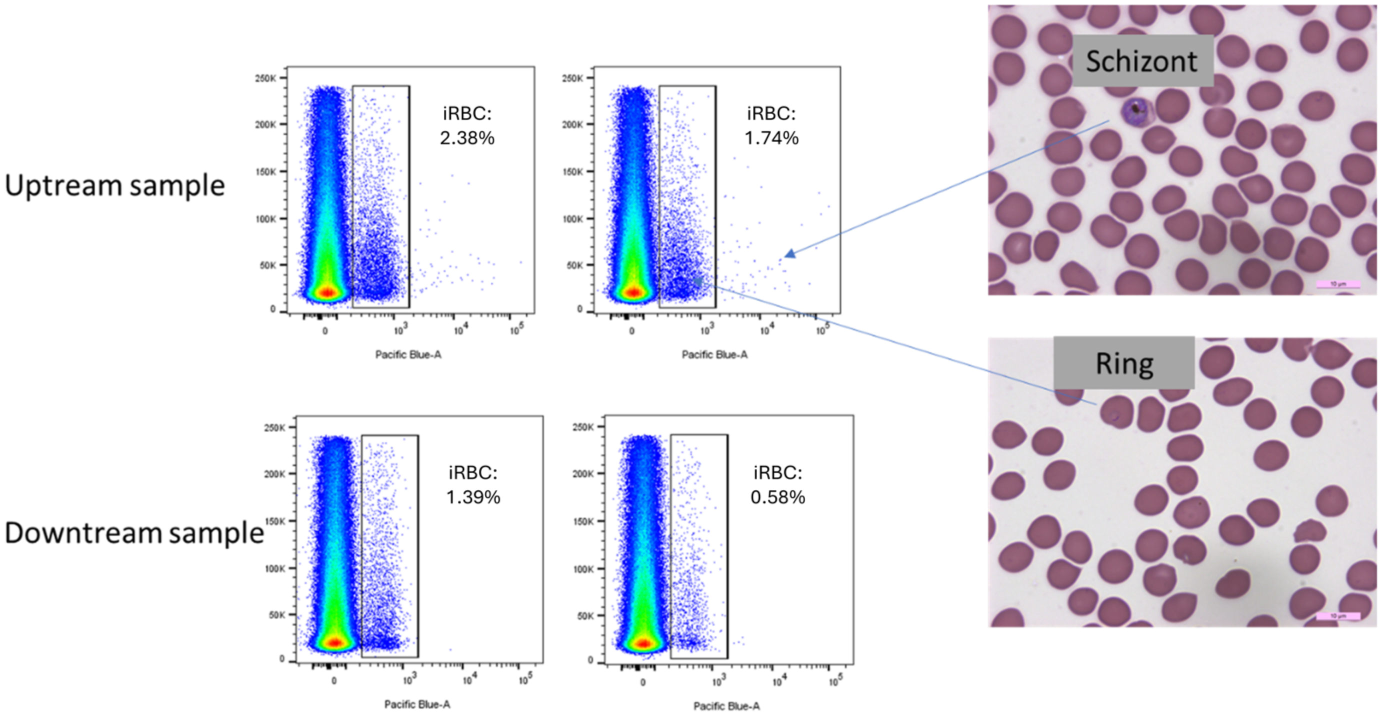

APPENDIX FIGURE S5. Parasite populations before and after microsphiltration. Shown are the input populations (top panels), which contain rings (indicated in the box) and schizonts (on the right-hand side of the panel). As the schizonts cannot pass through the microsphiltration column, only rings are detected in the flow-through sample. Indicated in each panel is the parasitemia ('iRBCs'). Note that no schizonts are present in the flow-through sample. This is confirmed with Giemsa-staining of thin blood films (right-hand panels).

APPENDIX TABLE S1. Statistical analysis of data in Figure 2B.

	20 min D	20 min R	30 min D	30 min R	1hpi D	1hpi R	2hpi D	2hpi R	4hpi D	4hpi R	6hpi D	6hpi R	16hpi D	16hpi R	24hpi D	24hpi R
**20 min D**																
**20 min R**	ns (0.4861)															
**30 min D**	ns (0.9999)															
**30 min R**		ns (0.9999)	<0.0001 (****)													
**1hpi D**	0.0051(**)		ns (0.8687)													
**1hpi R**		ns (0.9999)		ns (0.9999)	<0.0001 (****)											
**2hpi D**	<0.0001 (****)		0.0004 (***)		ns (0.9999)											
**2hpi R**		ns (0.9999)		ns (0.9999)		ns (0.9999)	<0.0001 (****)									
**4hpi D**	<0.0001 (****)		0.0093 (**)		ns (0.9999)		ns (0.9999)									
**4hpi R**		ns (0.9999)		ns (0.9999)		ns (0.9999)		ns (0.9999)	<0.0001 (****)							
**6hpi D**	<0.0001 (****)		0.0059 (**)		ns (0.9999)		ns (0.9999)		ns (0.9999)							
**6hpi R**		ns (0.9999)		ns (0.9999)		ns (0.9999)		ns (0.9999)		ns (0.9999)	<0.0001 (****)					
**16hpi D**	<0.0001 (****)		<0.0001 (****)		0.0009 (***)		ns (0.9999)		ns (0.1277)		ns (0.1382)					
**16hpi R**		ns (0.9999)		ns (0.6884)		0.0333 (*)		ns (0.7475)		ns (0.9999)		ns (0.9999)	<0.0001 (****)			
**24hpi D**	<0.0001 (****)		<0.0001 (****)		0.0006 (***)		ns (0.9999)		ns (0.1062)		ns (0.1147)		ns (0.9999)			
**24hpi R**		ns (0.9999)		ns (0.9999)		ns (0.9999)		ns (0.9999)		ns (0.9999)		ns (0.9999)		ns (0.1743)	<0.0001 (****)	

APPENDIX TABLE S2. Comparison of surface area of uninfected erythrocytes (uRBC) and erythrocytes infected with DMSO-treated PV6 DiCre parasite (iRBC). Time represents time after removal of egress inhibitor.

			Average surface area (μm^2)		Difference uRBC vs iRBC	
Time	Sample	Strain	uRBC	iRBC	Area (μm^2)	%
30	1	PV6 diCre (529)	65.6	67.52	-1.92	-2.9
	2	PV6 diCre (529)	64.87	68.42	-3.55	-5.47
	3	PV6 DiCre (529)	83.34	77.87	5.47	6.6
	4	PV6 DiCre (A8)	78.49	74.66	3.83	4.9
90	1	PV6 DiCre (529)	84.38	80.49	3.89	4.6
	2	PV6 DiCre (A8)	77.93	75.15	2.78	3.6
Average (Std Dev)					1.54	1.88 (4.88)
