# Supplementary figures and images for: Malaria parasites undergo a rapid and extensive metamorphosis after invasion of the host erythrocyte
Source: EMBO Rep. 2025 Apr 4;26(10):2545–73. doi: 10.1038/s44319-025-00435-3 (PMC12116788; doi:10.1038/s44319-025-00435-3)

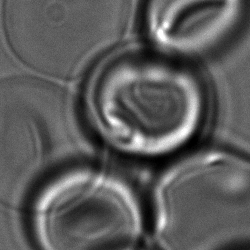

Supplement: Supplementary file 12 — Source data Fig. 1 [file 44319_2025_435_MOESM12_ESM.zip › 1+/1G+/ndseq4009-0001white.tif]

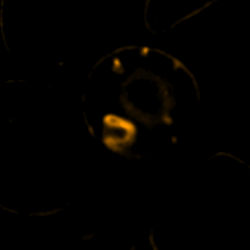

Supplement: Supplementary file 12 — Source data Fig. 1 [file 44319_2025_435_MOESM12_ESM.zip › 1+/1G+/ndseq4009-0001orangeRGB.tif]

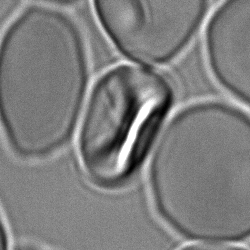

Supplement: Supplementary file 12 — Source data Fig. 1 [file 44319_2025_435_MOESM12_ESM.zip › 1+/1G+/Pk amoeboid white ndseq28536_crop-0017RGB320.tif]

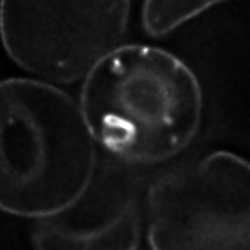

Supplement: Supplementary file 12 — Source data Fig. 1 [file 44319_2025_435_MOESM12_ESM.zip › 1+/1G+/ndseq4009-0001orange.tif]

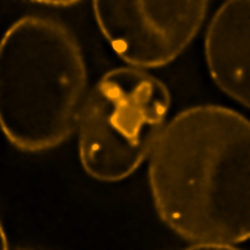

Supplement: Supplementary file 12 — Source data Fig. 1 [file 44319_2025_435_MOESM12_ESM.zip › 1+/1G+/Pk amoeboid orange ndseq28536_crop-0015RGB.tif]

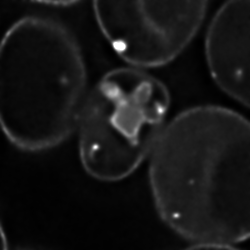

Supplement: Supplementary file 12 — Source data Fig. 1 [file 44319_2025_435_MOESM12_ESM.zip › 1+/1G+/Pk amoeboid orange ndseq28536_crop-0015.tif]

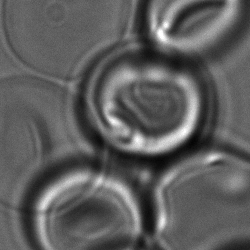

Supplement: Supplementary file 12 — Source data Fig. 1 [file 44319_2025_435_MOESM12_ESM.zip › 1+/1G+/ndseq4009-0001whiteRGB.tif]

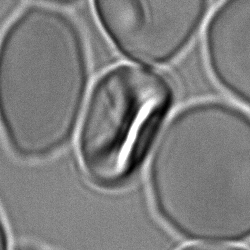

Supplement: Supplementary file 12 — Source data Fig. 1 [file 44319_2025_435_MOESM12_ESM.zip › 1+/1G+/Pk amoeboid white ndseq28536_crop-0017RGB.tif]

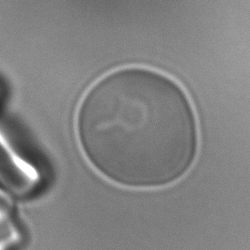

Supplement: Supplementary file 12 — Source data Fig. 1 [file 44319_2025_435_MOESM12_ESM.zip › 1+/1A+/DMSO 5 flaskRGB.tif]

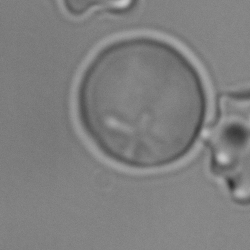

Supplement: Supplementary file 12 — Source data Fig. 1 [file 44319_2025_435_MOESM12_ESM.zip › 1+/1A+/DMSO 1 amoeboid crop 320.tif]

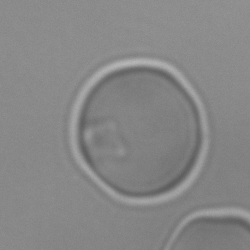

Supplement: Supplementary file 12 — Source data Fig. 1 [file 44319_2025_435_MOESM12_ESM.zip › 1+/1A+/DMSO 4 squareRGB.tif]

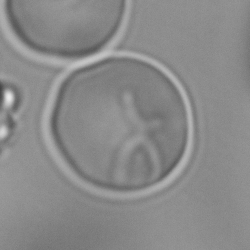

Supplement: Supplementary file 12 — Source data Fig. 1 [file 44319_2025_435_MOESM12_ESM.zip › 1+/1E+/HBBS crop 320.tif]

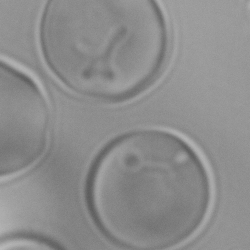

Supplement: Supplementary file 12 — Source data Fig. 1 [file 44319_2025_435_MOESM12_ESM.zip › 1+/1E+/cyclo crop 320.tif]

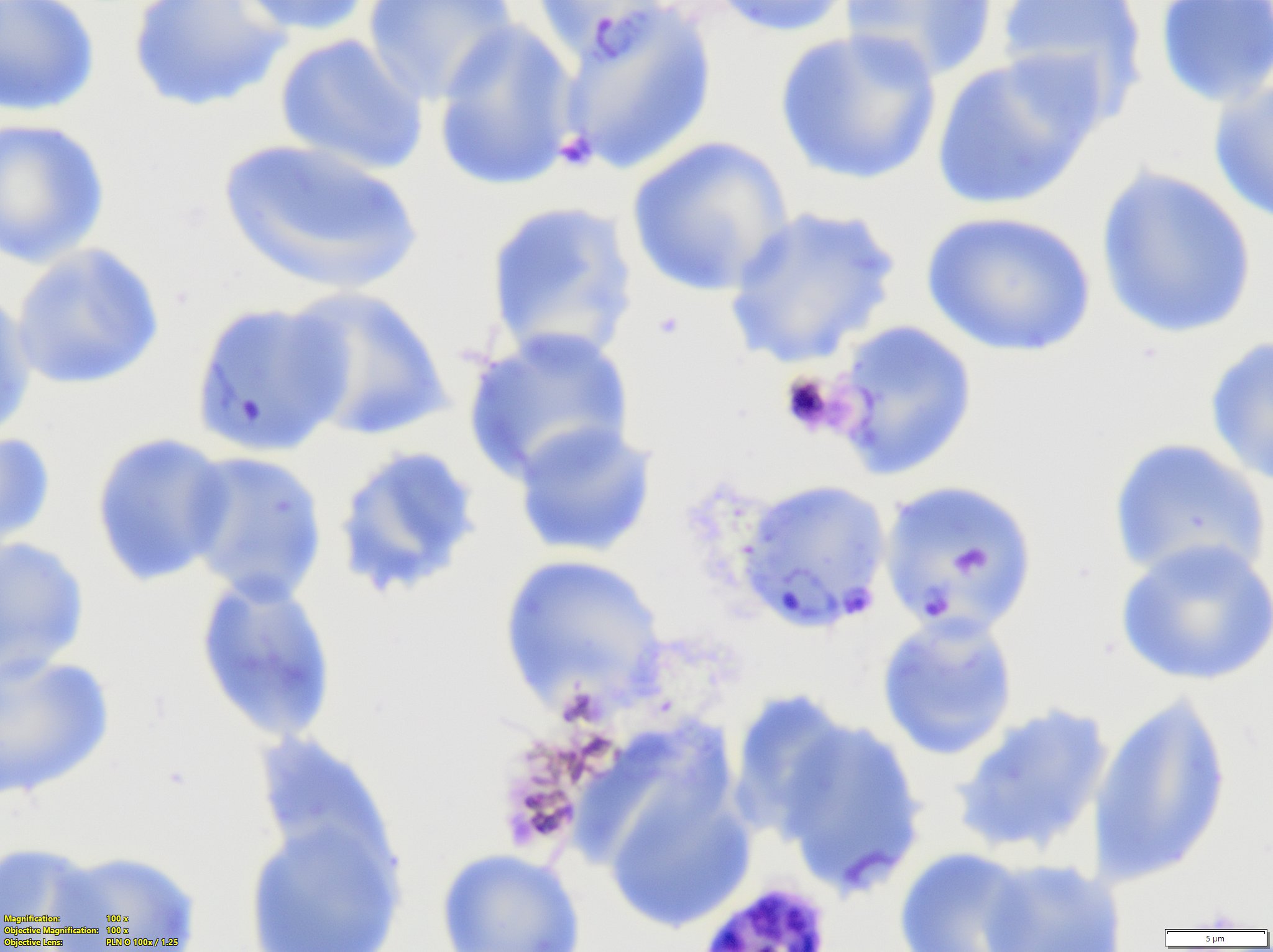

Supplement: Supplementary file 13 — Source data Fig. 2 [file 44319_2025_435_MOESM13_ESM.zip › 2+/2A +/DMSO_30mins.jpg]

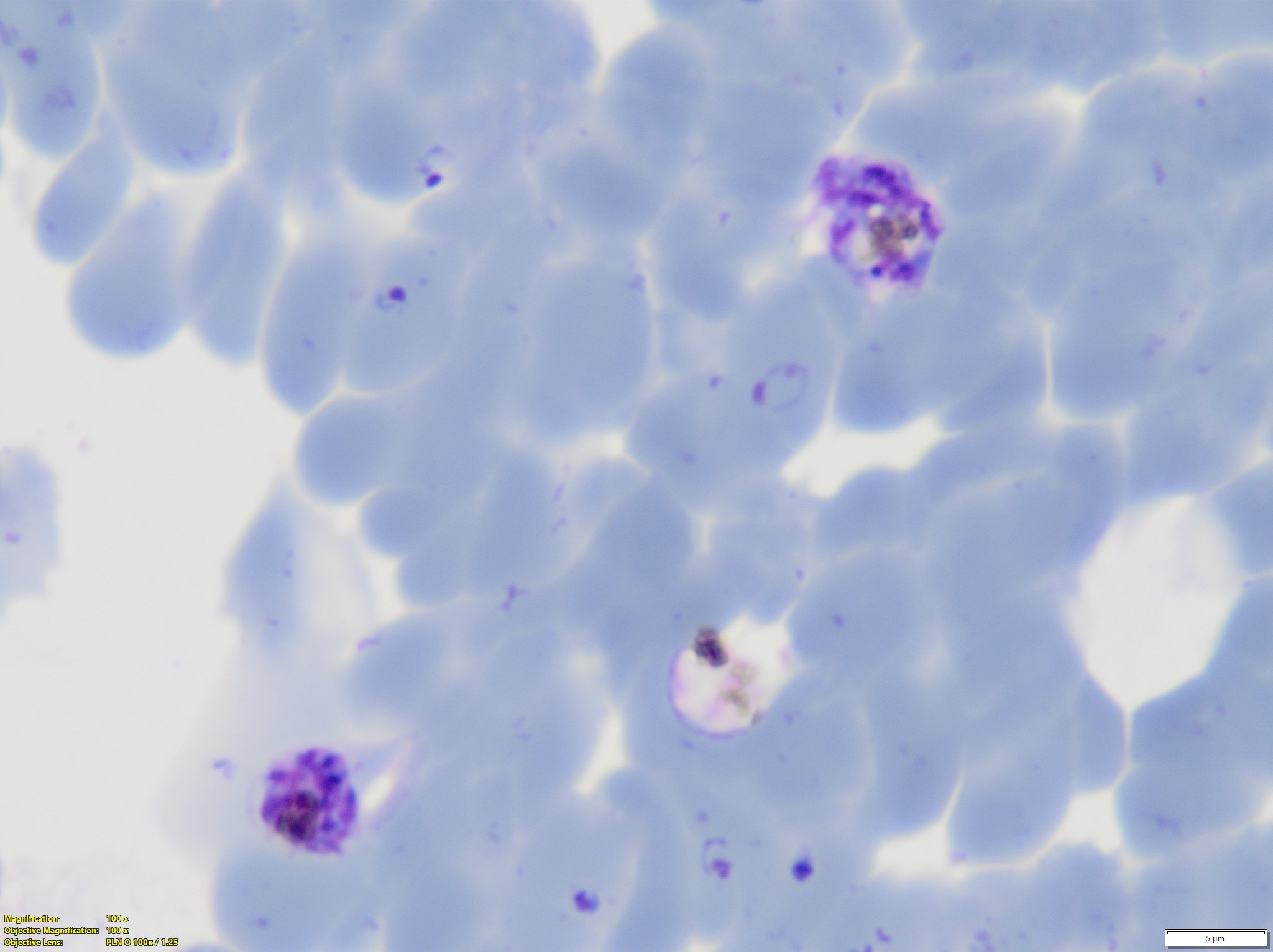

Supplement: Supplementary file 13 — Source data Fig. 2 [file 44319_2025_435_MOESM13_ESM.zip › 2+/2A +/DMSO_20mins.jpg]

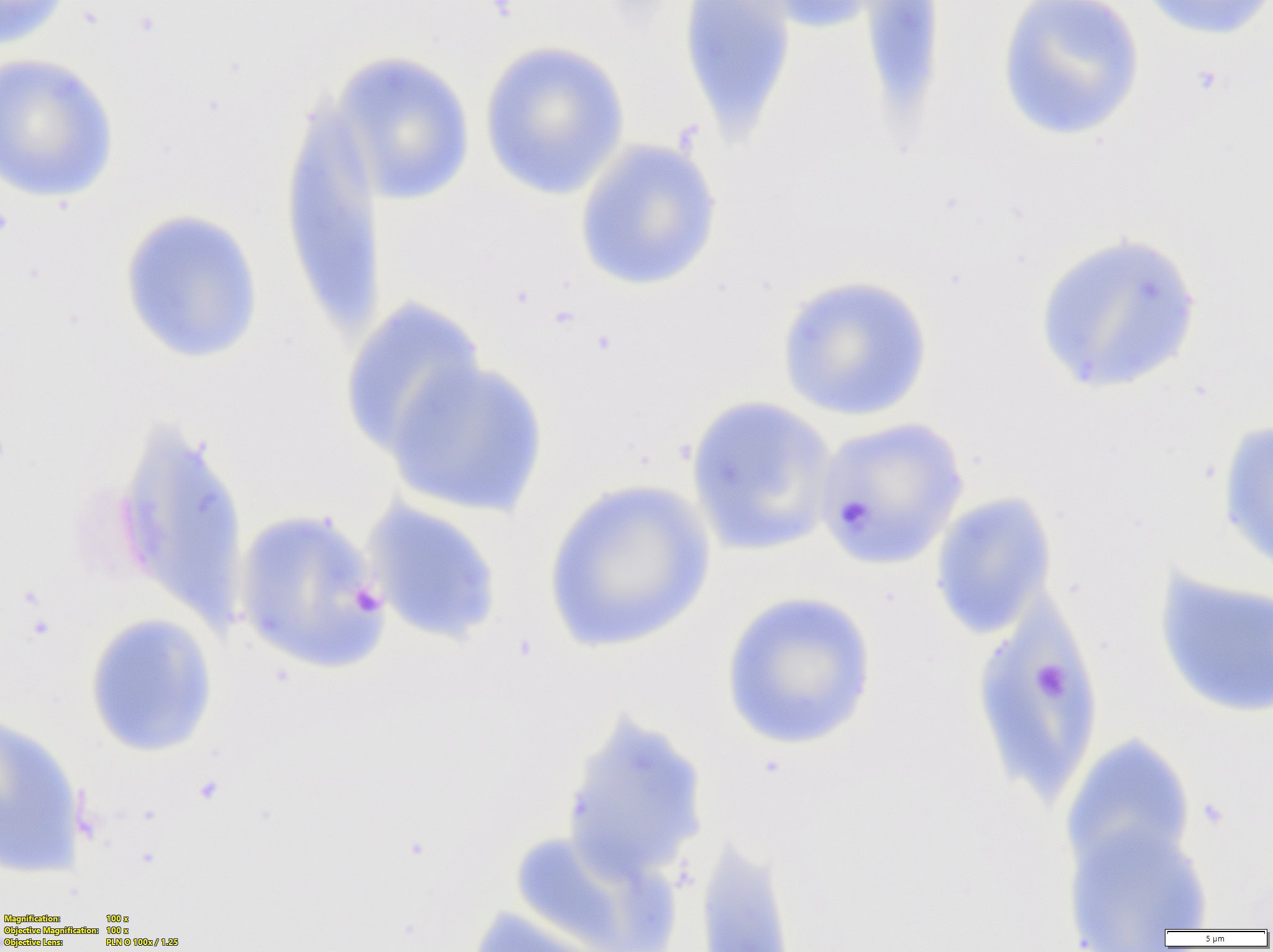

Supplement: Supplementary file 13 — Source data Fig. 2 [file 44319_2025_435_MOESM13_ESM.zip › 2+/2A +/RAPA_16h.jpg]

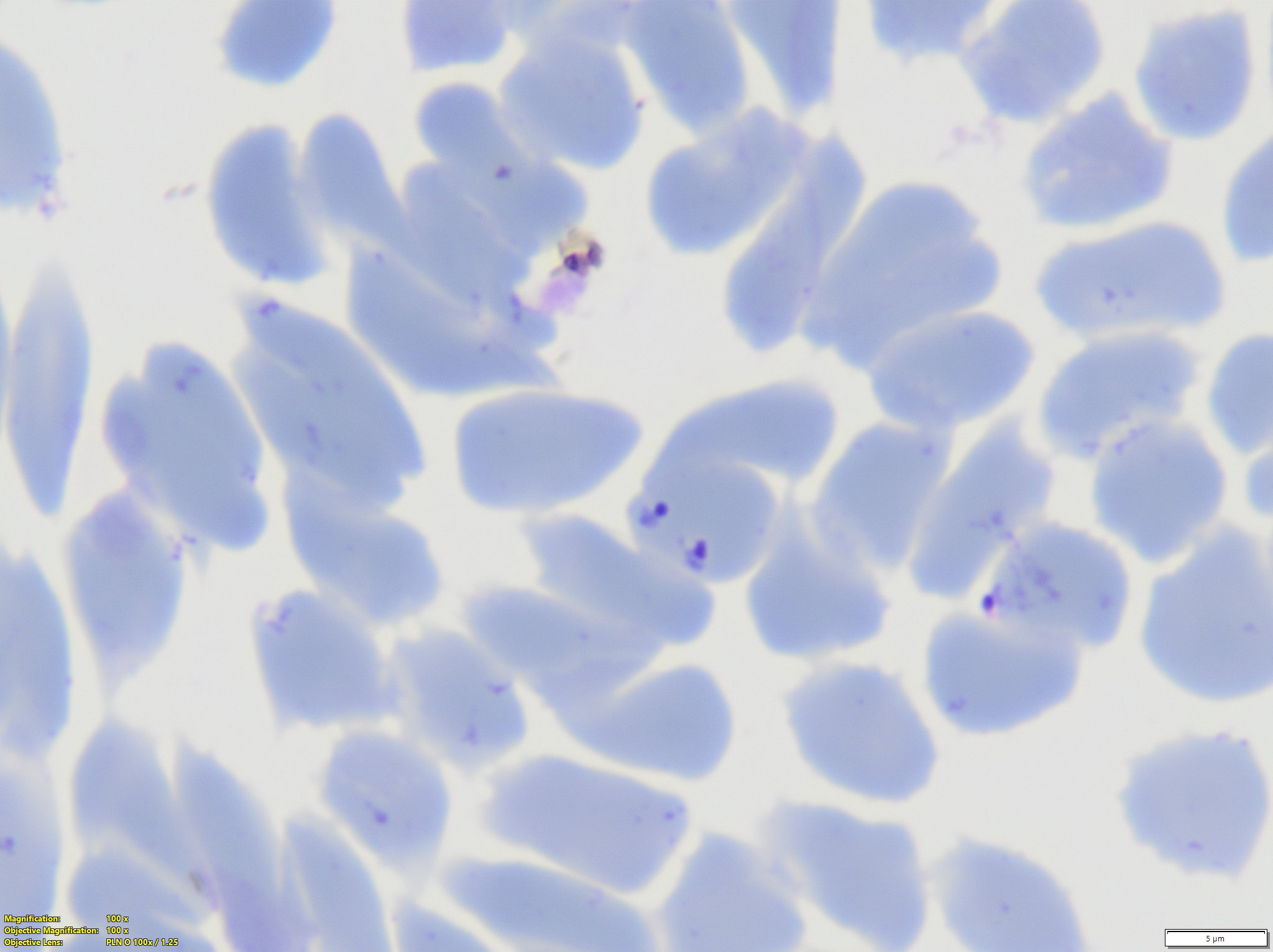

Supplement: Supplementary file 13 — Source data Fig. 2 [file 44319_2025_435_MOESM13_ESM.zip › 2+/2A +/RAPA_2h.jpg]

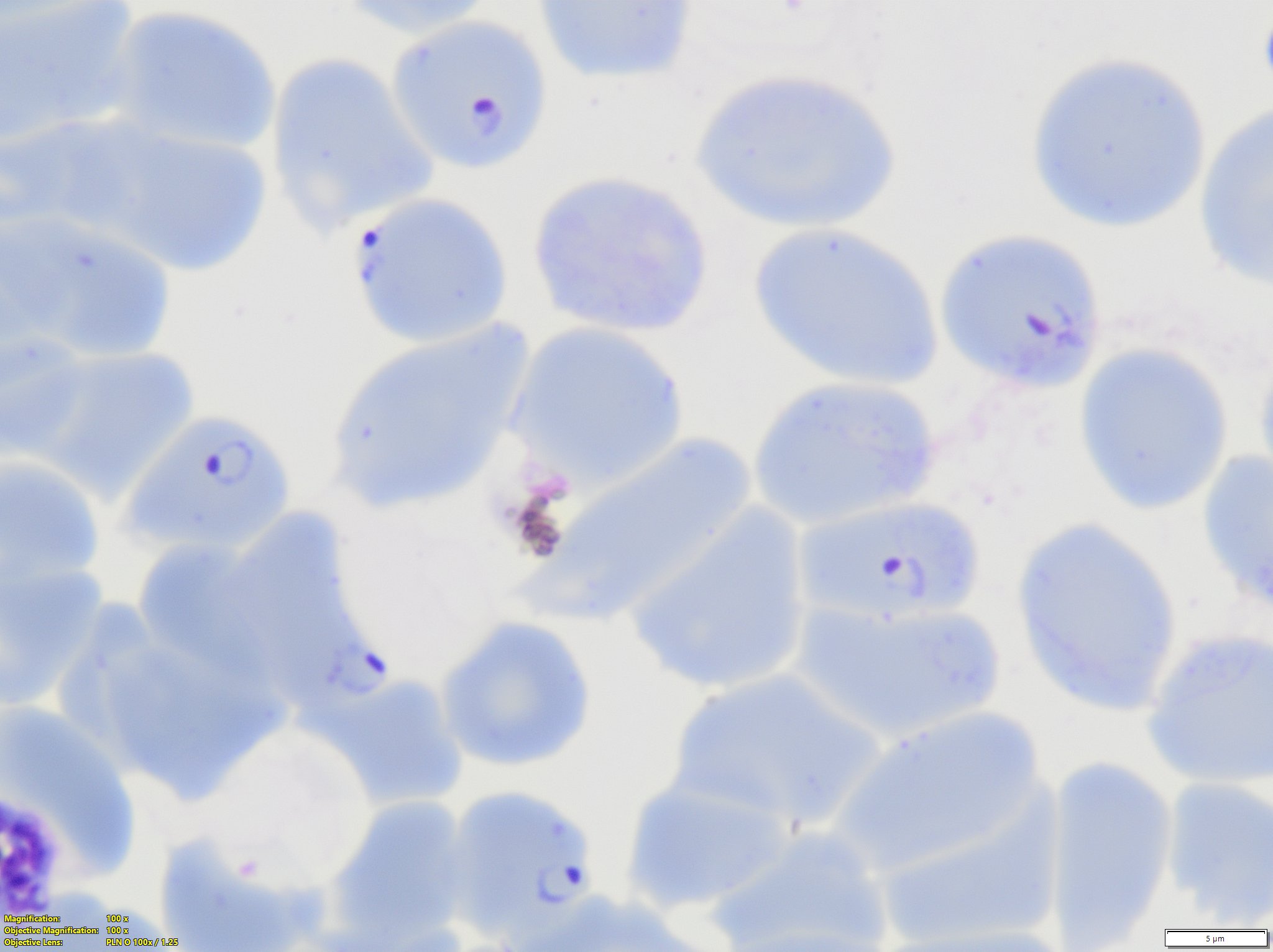

Supplement: Supplementary file 13 — Source data Fig. 2 [file 44319_2025_435_MOESM13_ESM.zip › 2+/2A +/DMSO_1h.jpg]

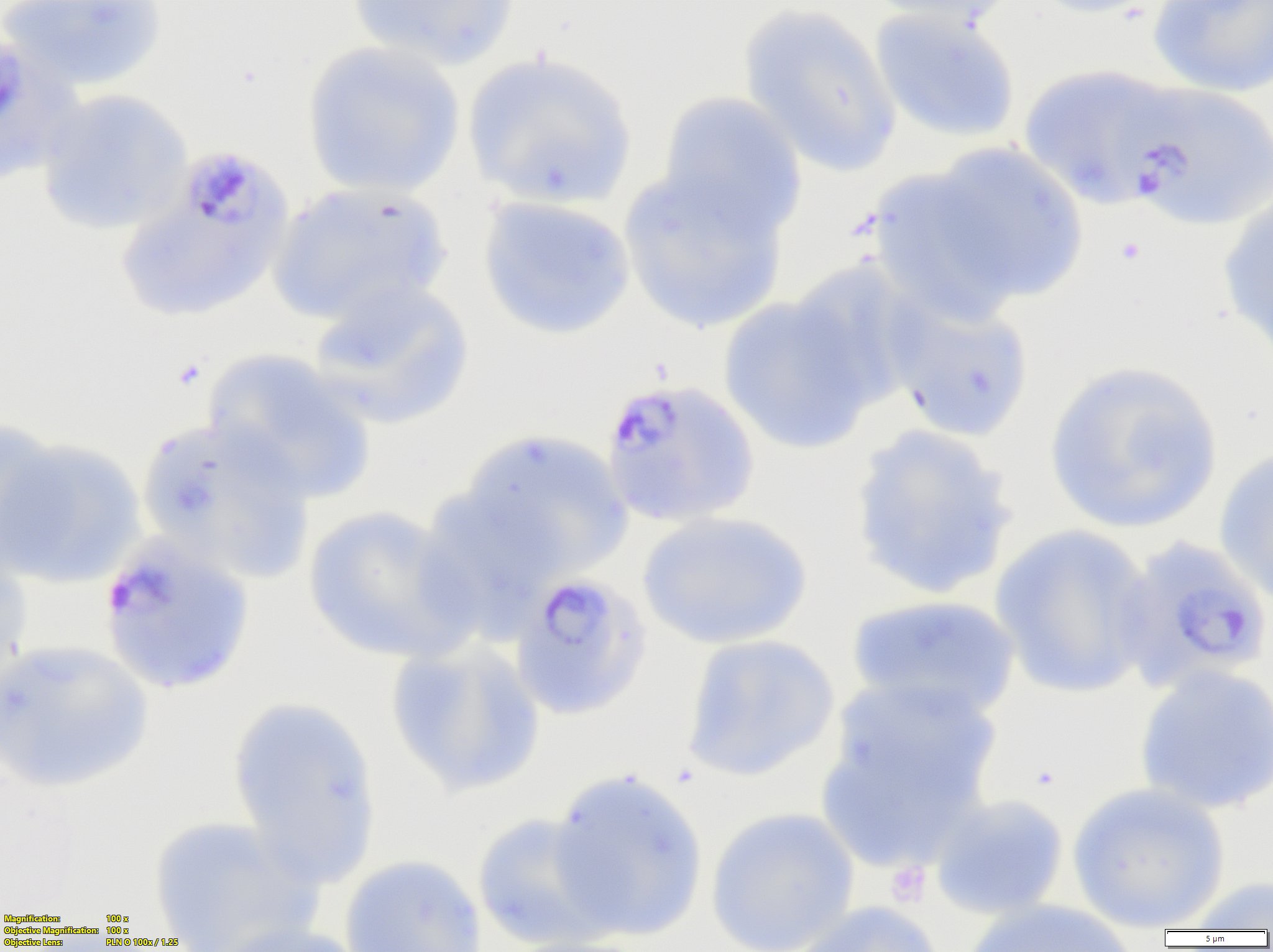

Supplement: Supplementary file 13 — Source data Fig. 2 [file 44319_2025_435_MOESM13_ESM.zip › 2+/2A +/DMSO_16h.jpg]

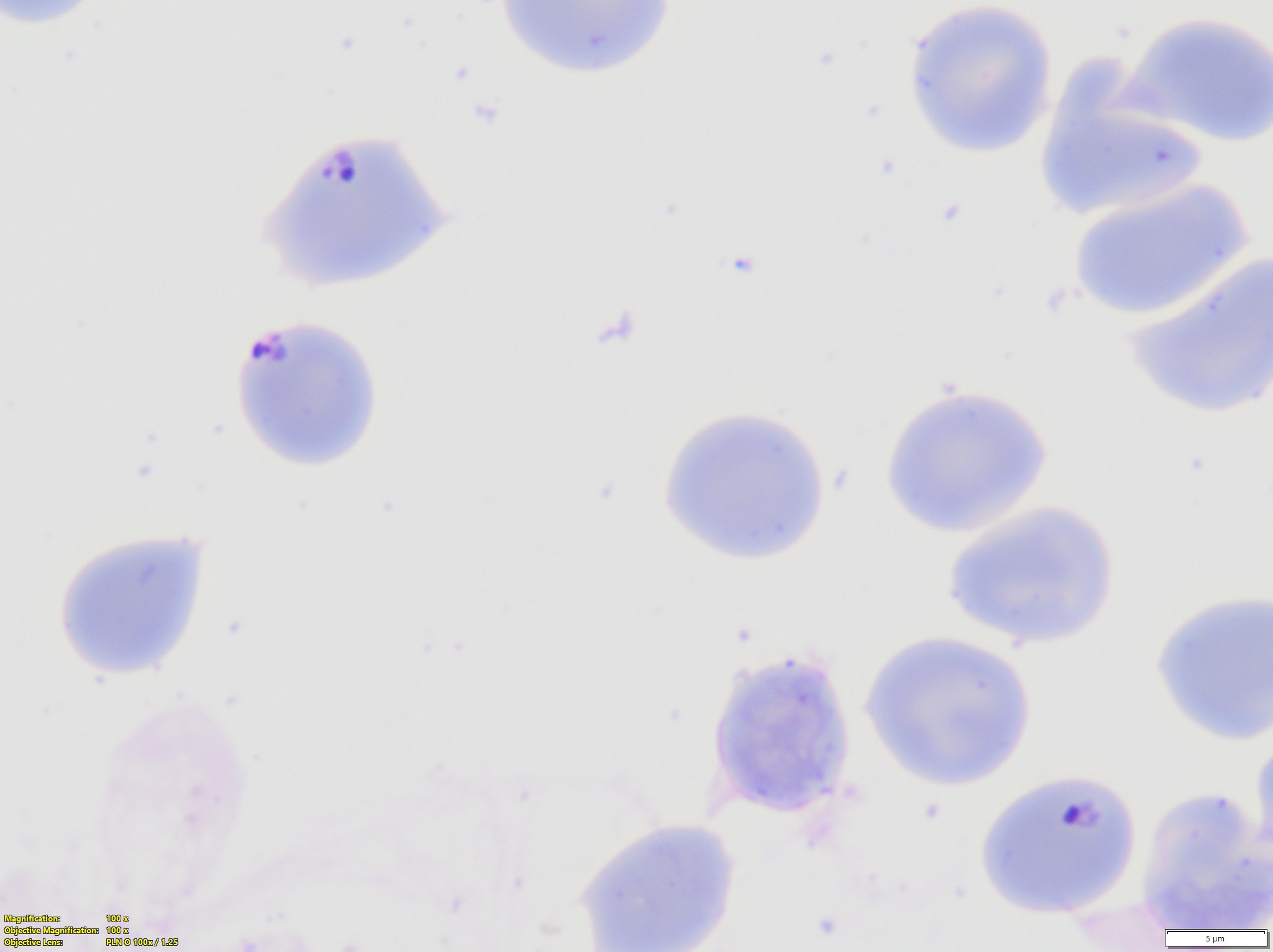

Supplement: Supplementary file 13 — Source data Fig. 2 [file 44319_2025_435_MOESM13_ESM.zip › 2+/2A +/RAPA_4h.jpg]

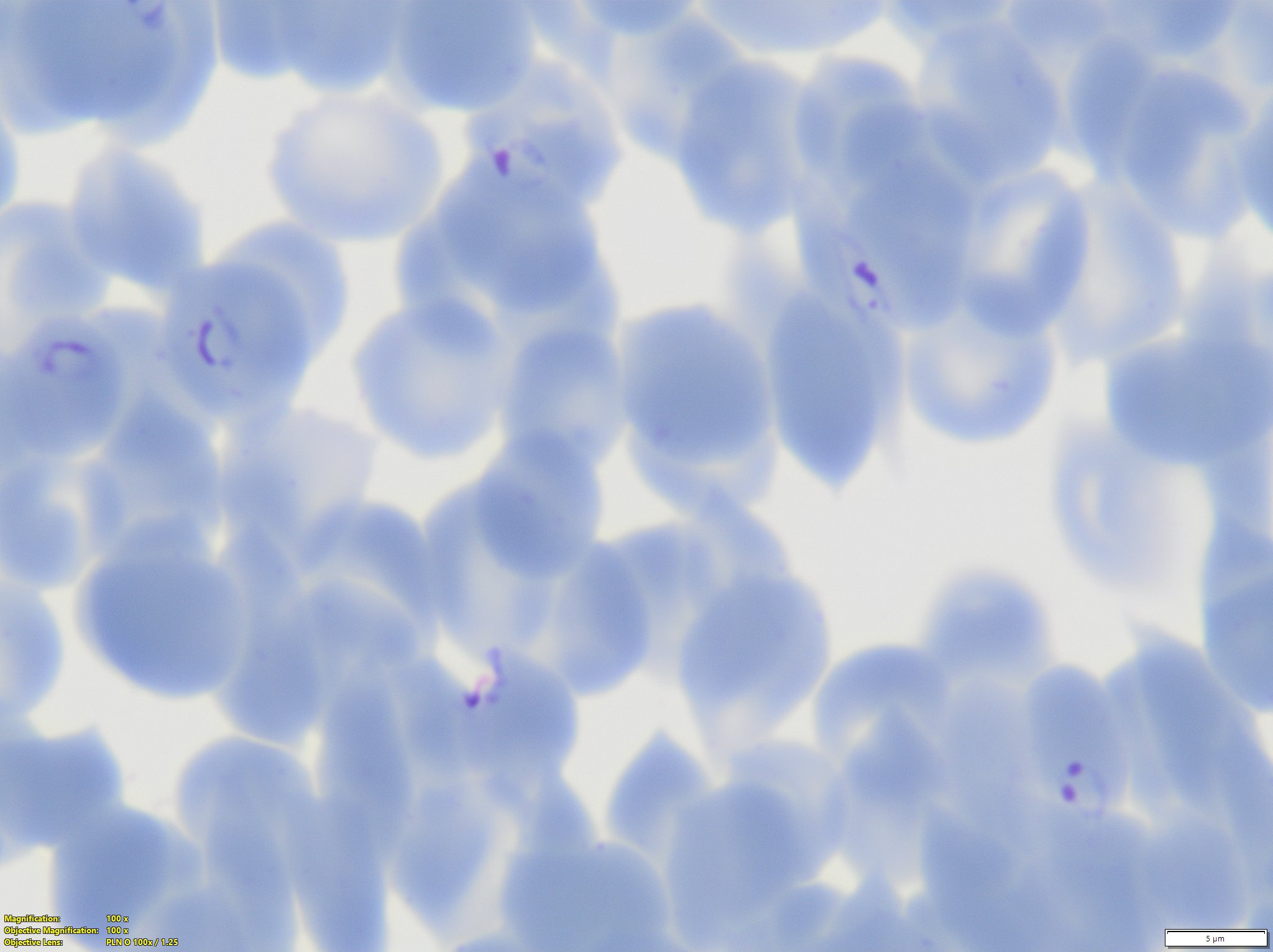

Supplement: Supplementary file 13 — Source data Fig. 2 [file 44319_2025_435_MOESM13_ESM.zip › 2+/2A +/DMSO_6h.jpg]

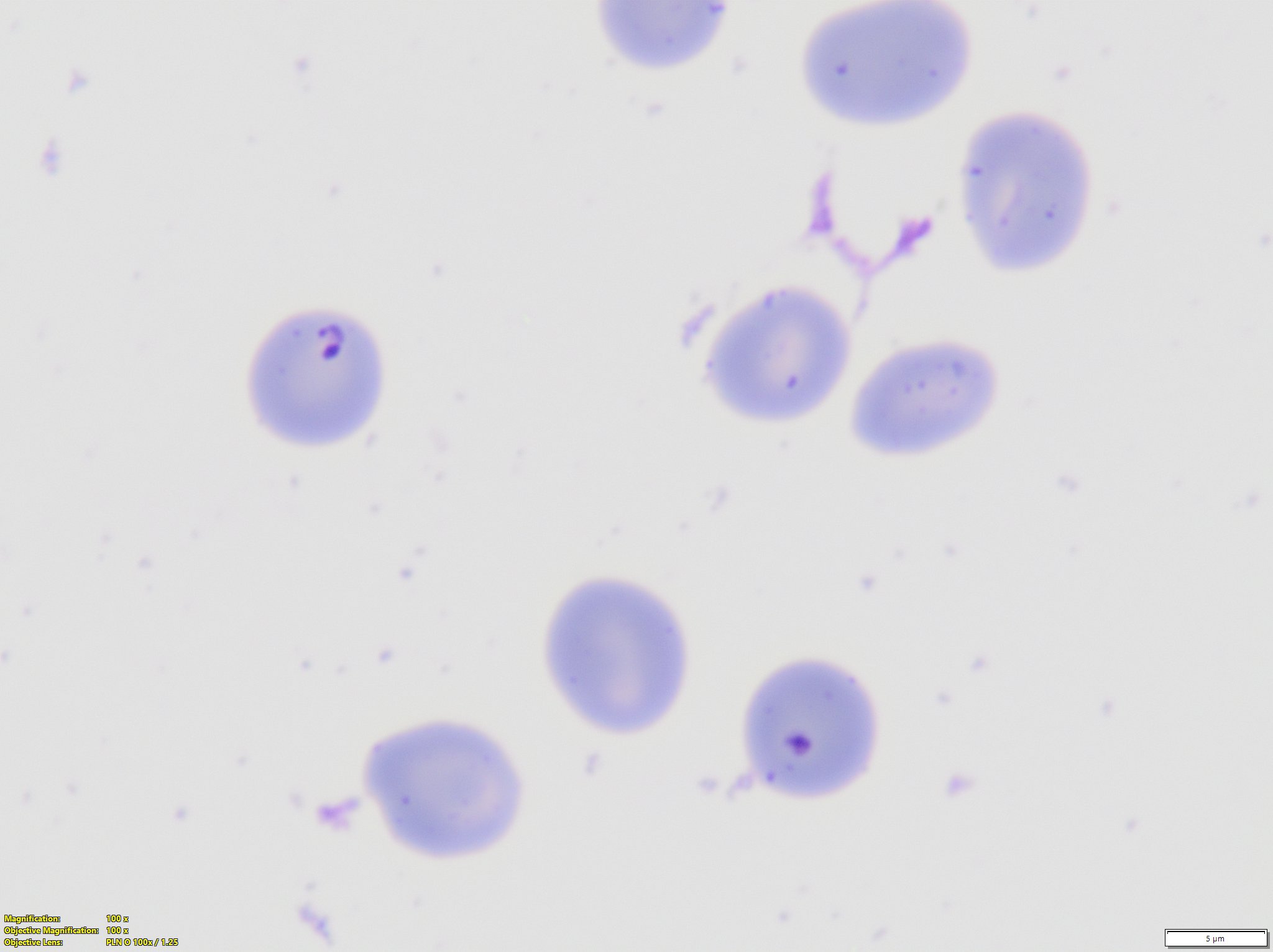

Supplement: Supplementary file 13 — Source data Fig. 2 [file 44319_2025_435_MOESM13_ESM.zip › 2+/2A +/RAPA_6h.jpg]

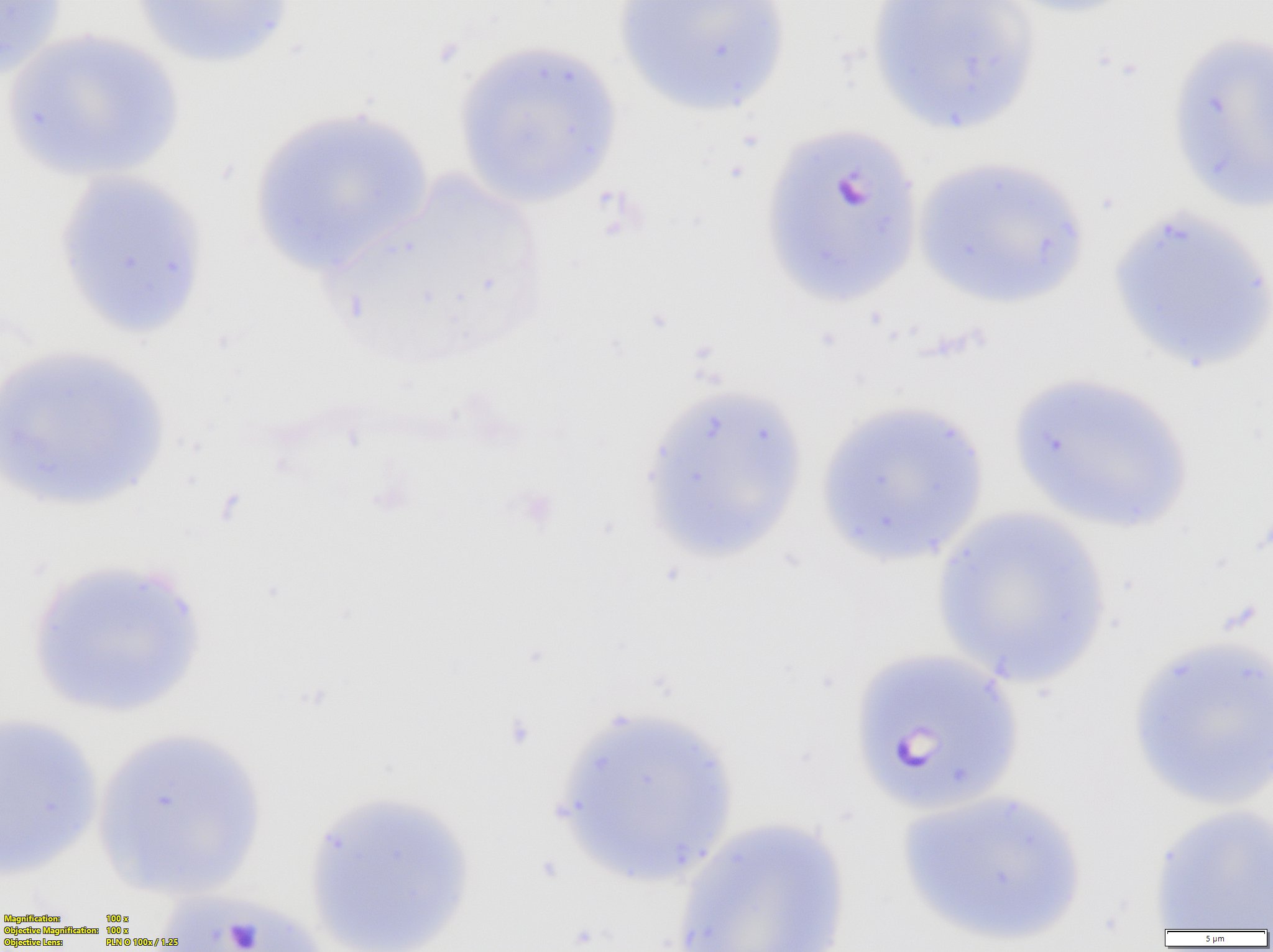

Supplement: Supplementary file 13 — Source data Fig. 2 [file 44319_2025_435_MOESM13_ESM.zip › 2+/2A +/RAPA_24h.jpg]

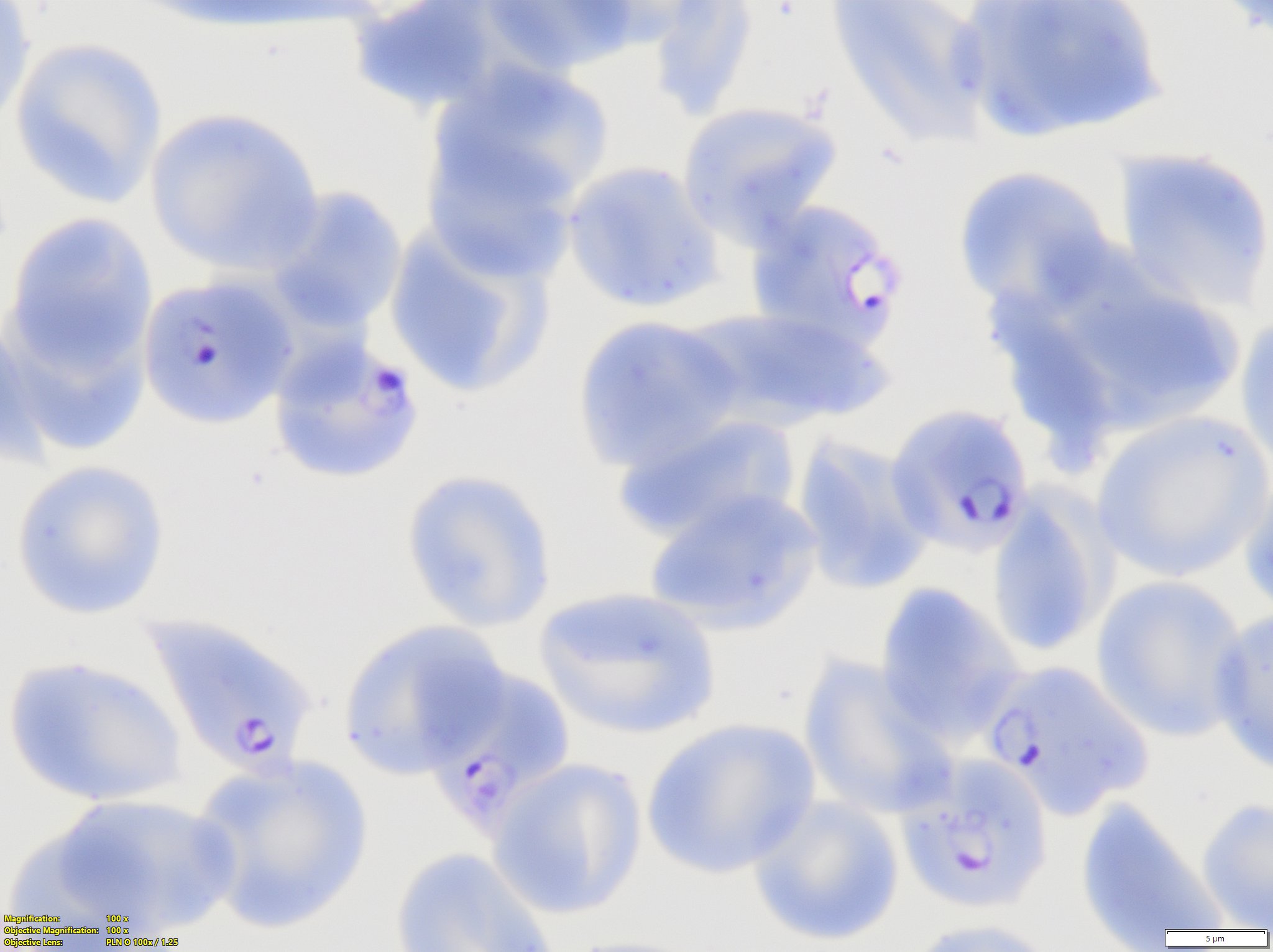

Supplement: Supplementary file 13 — Source data Fig. 2 [file 44319_2025_435_MOESM13_ESM.zip › 2+/2A +/DMSO_4h.jpg]

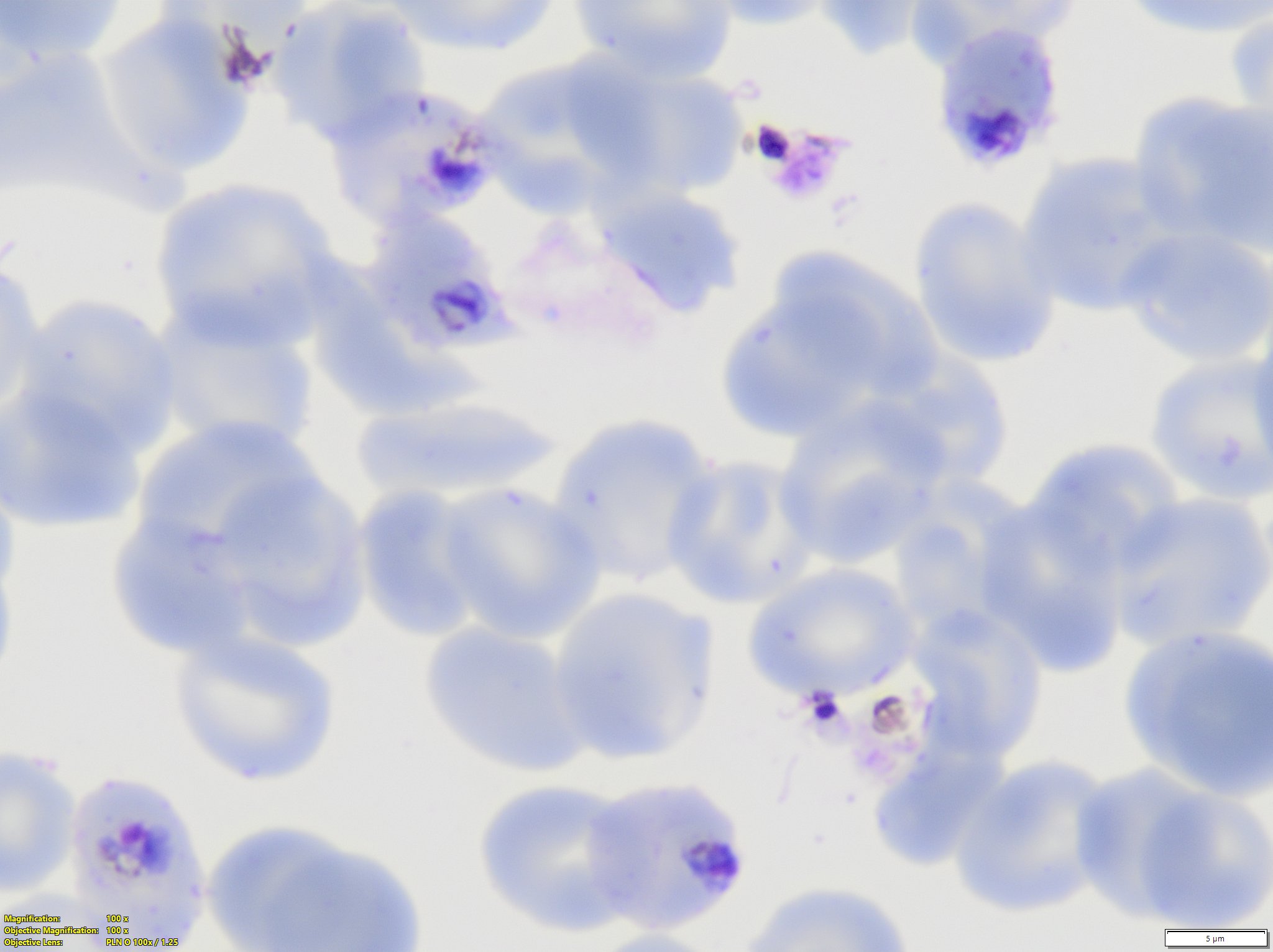

Supplement: Supplementary file 13 — Source data Fig. 2 [file 44319_2025_435_MOESM13_ESM.zip › 2+/2A +/DMSO_24h.jpg]

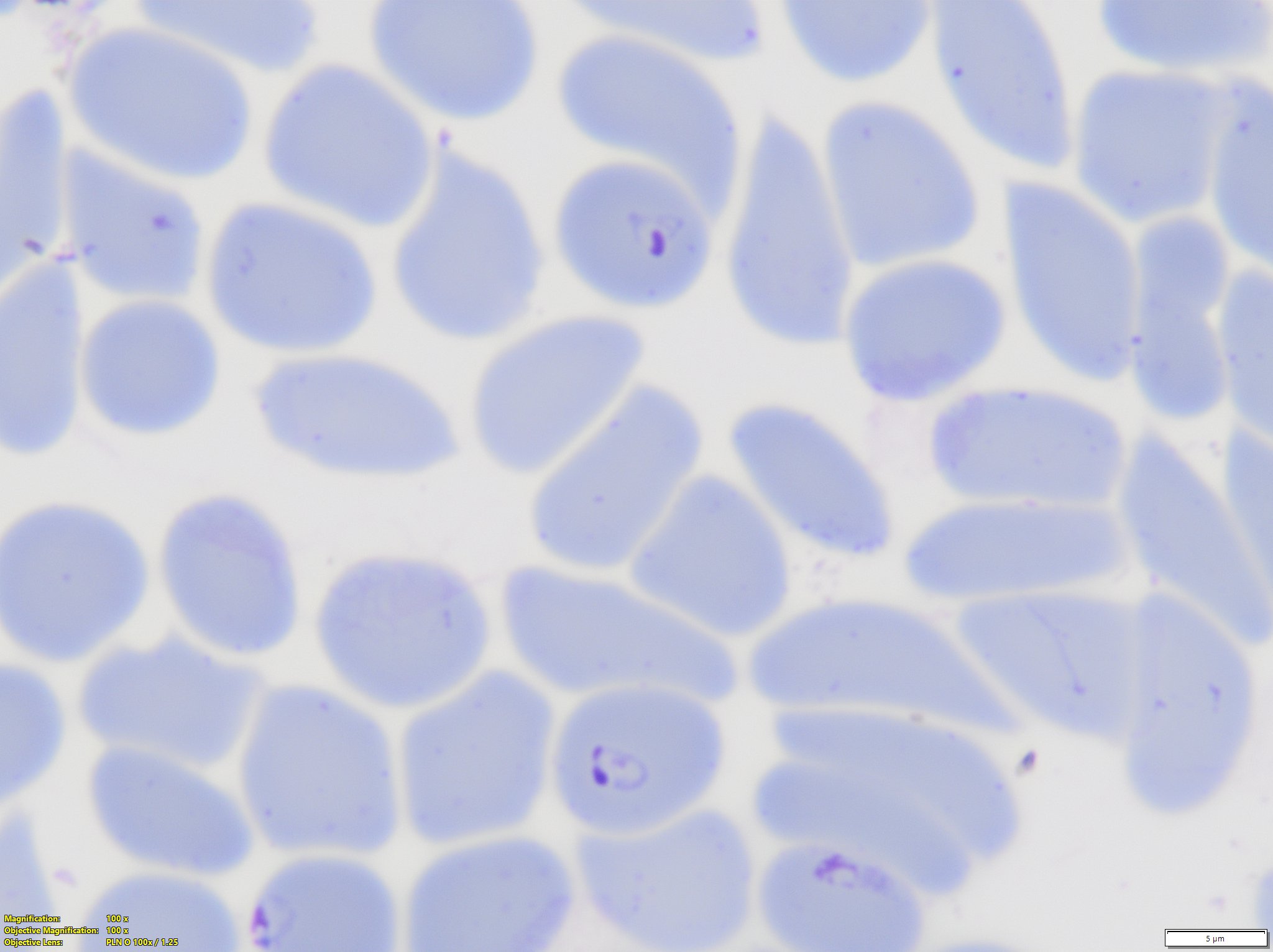

Supplement: Supplementary file 13 — Source data Fig. 2 [file 44319_2025_435_MOESM13_ESM.zip › 2+/2A +/DMSO_2h.jpg]

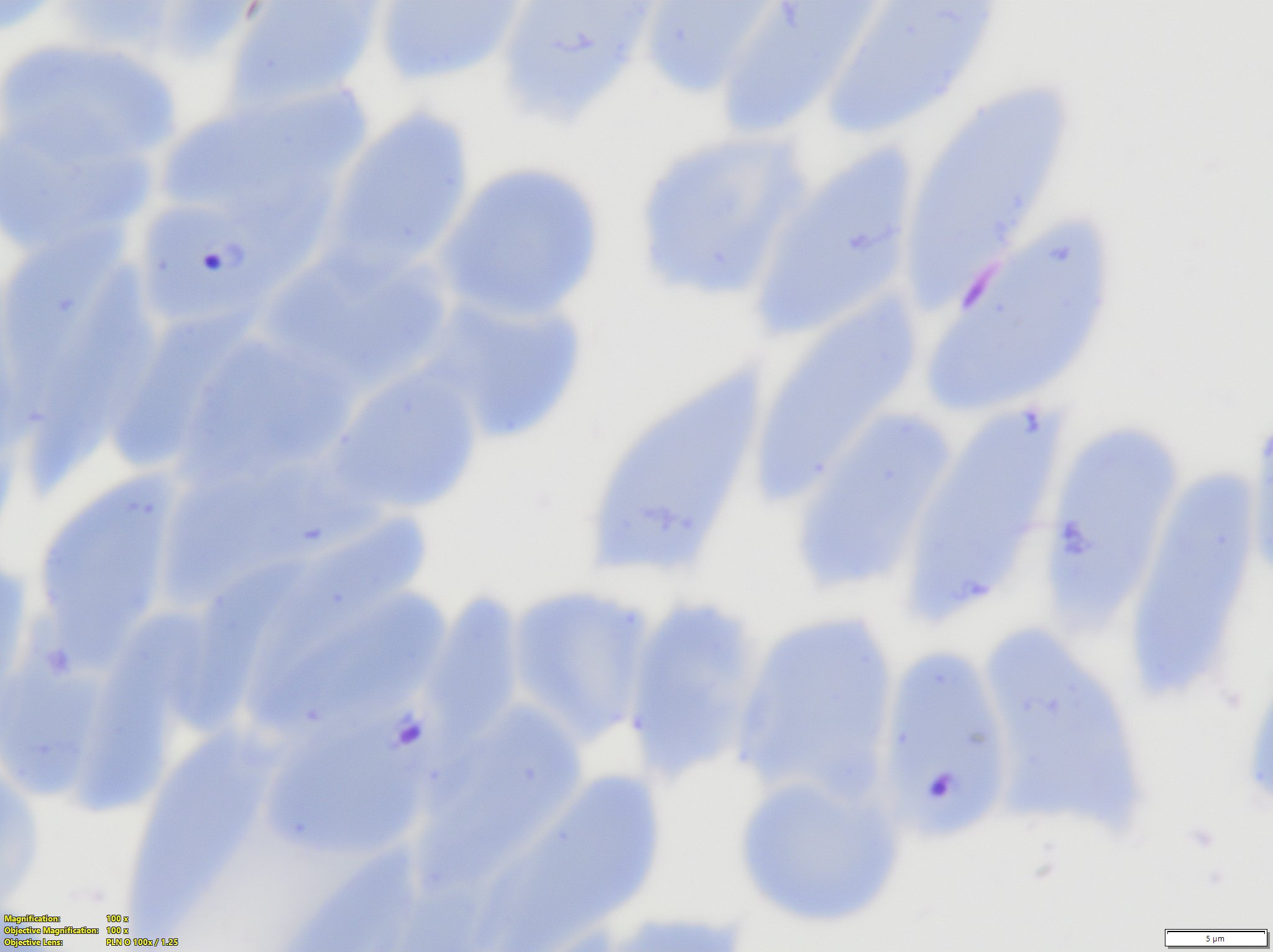

Supplement: Supplementary file 13 — Source data Fig. 2 [file 44319_2025_435_MOESM13_ESM.zip › 2+/2A +/RAPA_1h.jpg]

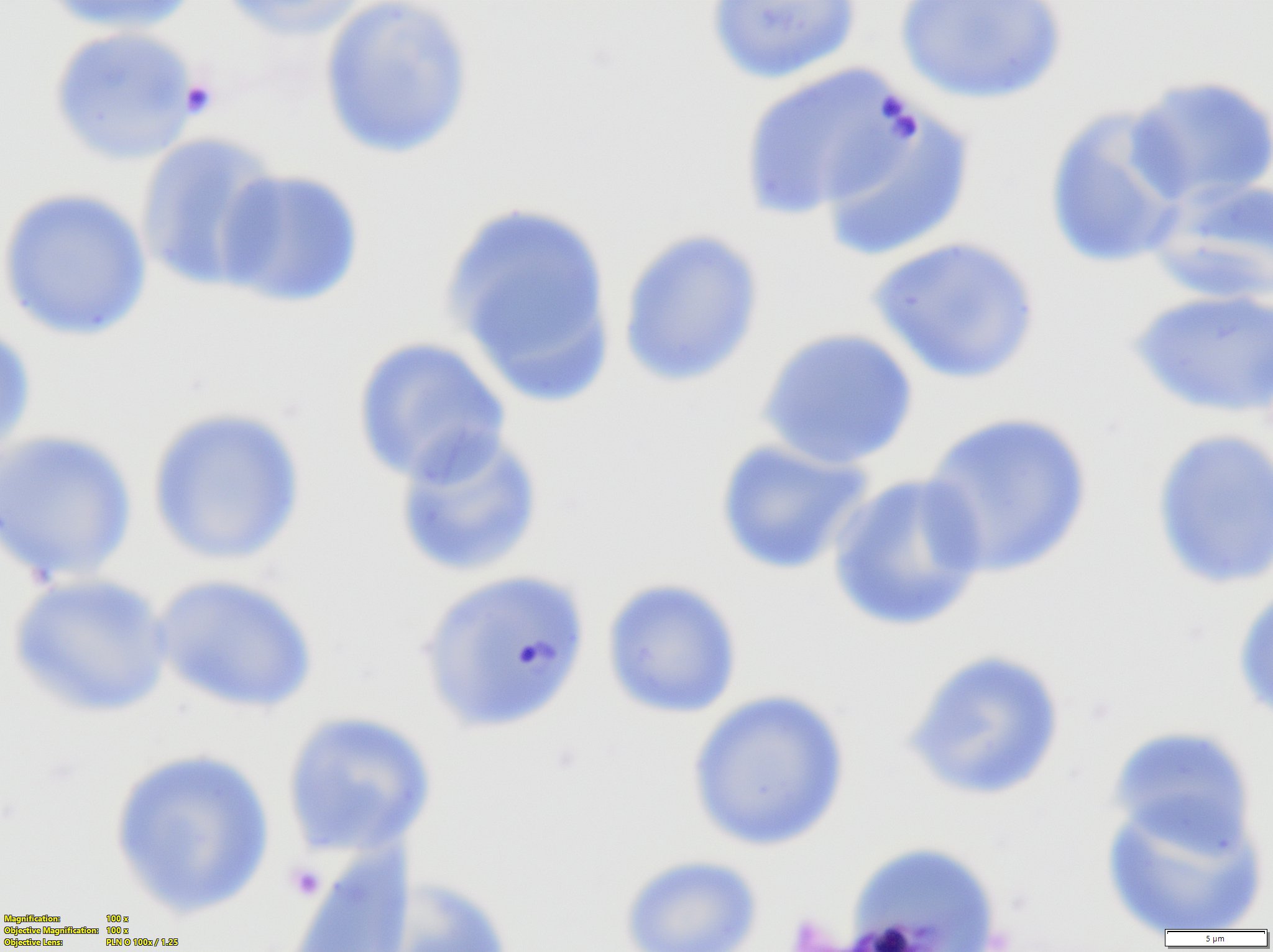

Supplement: Supplementary file 13 — Source data Fig. 2 [file 44319_2025_435_MOESM13_ESM.zip › 2+/2A +/RAPA_30mins.jpg]

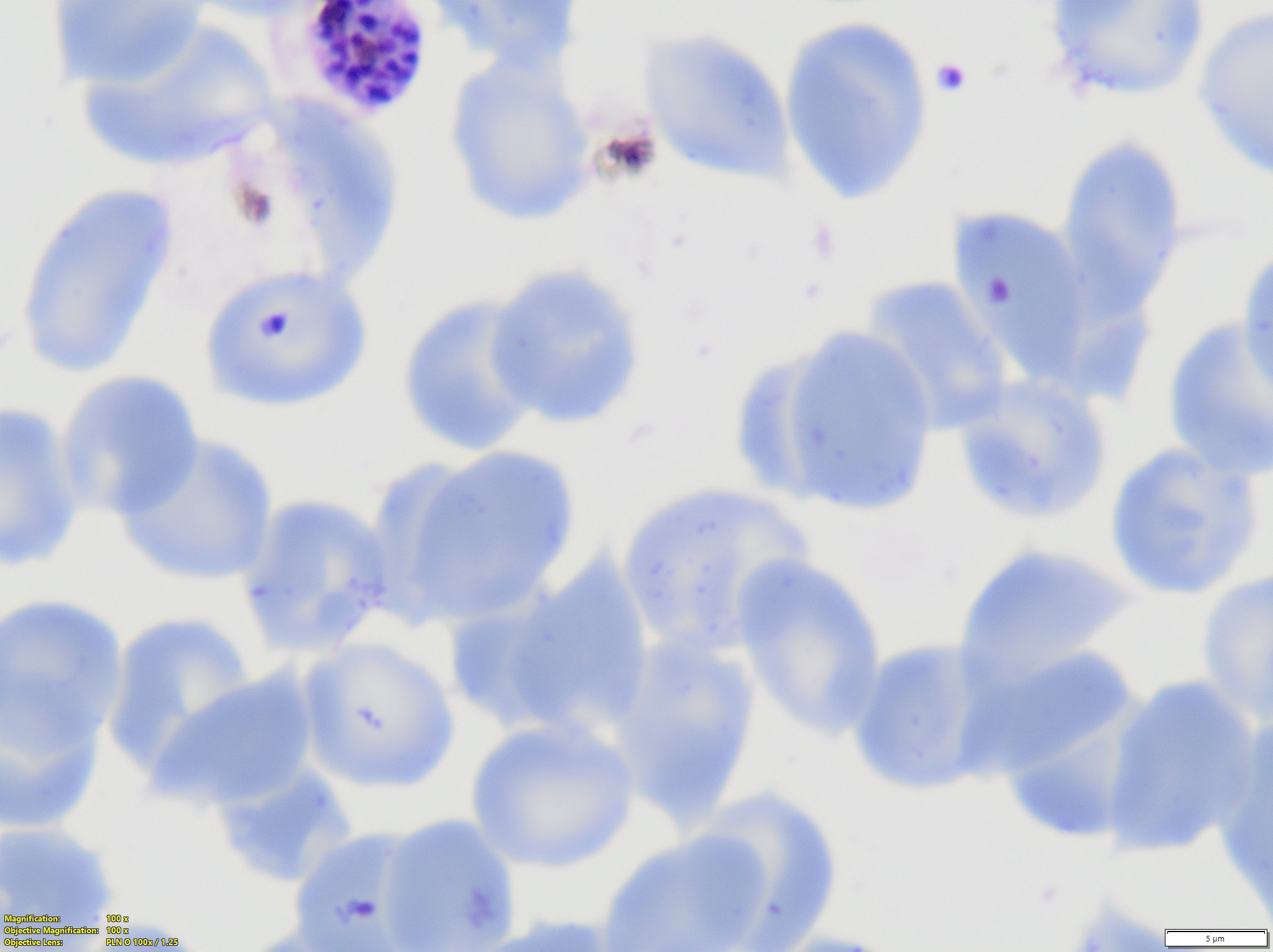

Supplement: Supplementary file 13 — Source data Fig. 2 [file 44319_2025_435_MOESM13_ESM.zip › 2+/2A +/RAPA_20mins.jpg]

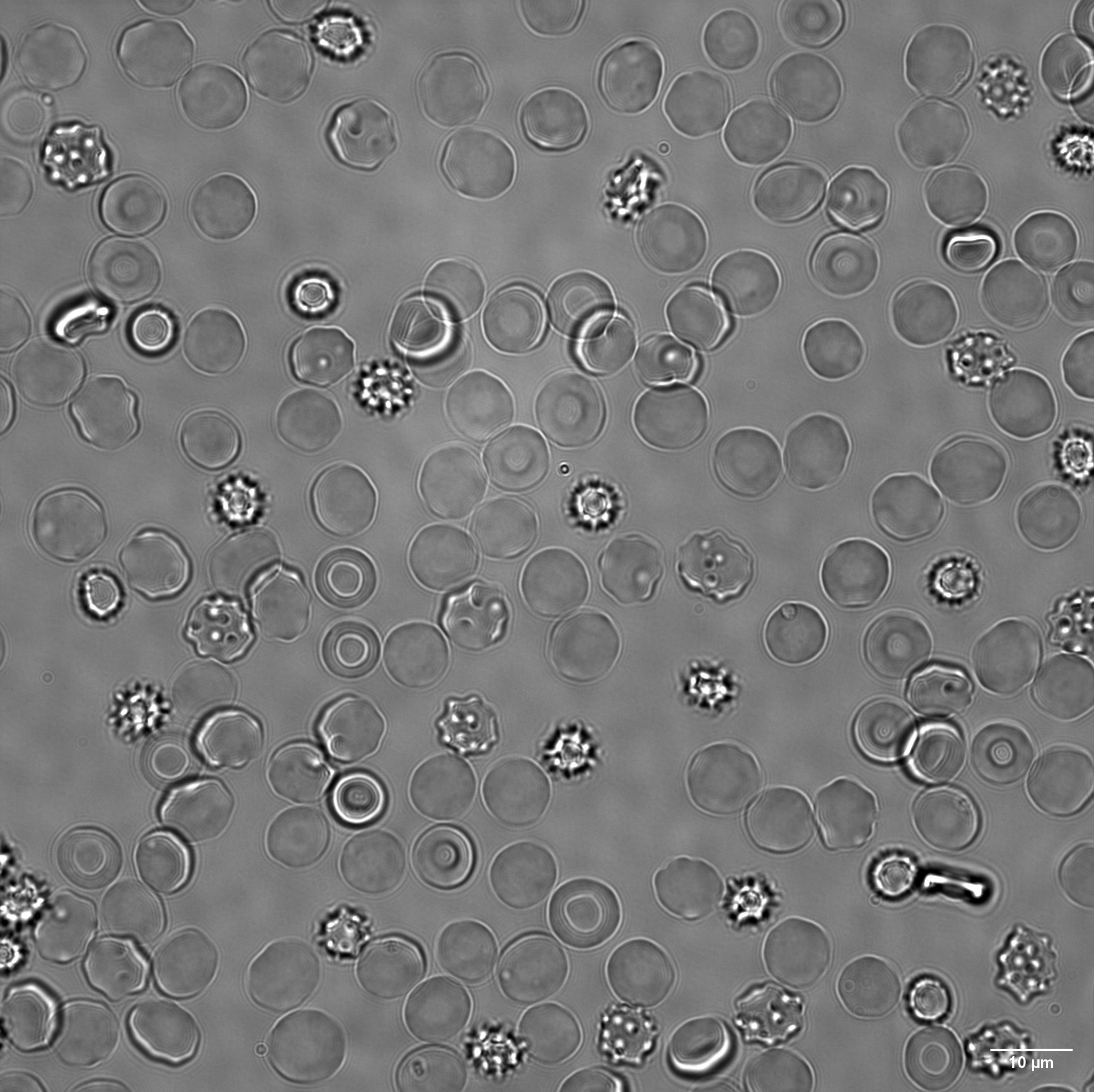

Supplement: Supplementary file 13 — Source data Fig. 2 [file 44319_2025_435_MOESM13_ESM.zip › 2+/2C +/Live_RAPA_2hpi.jpg]

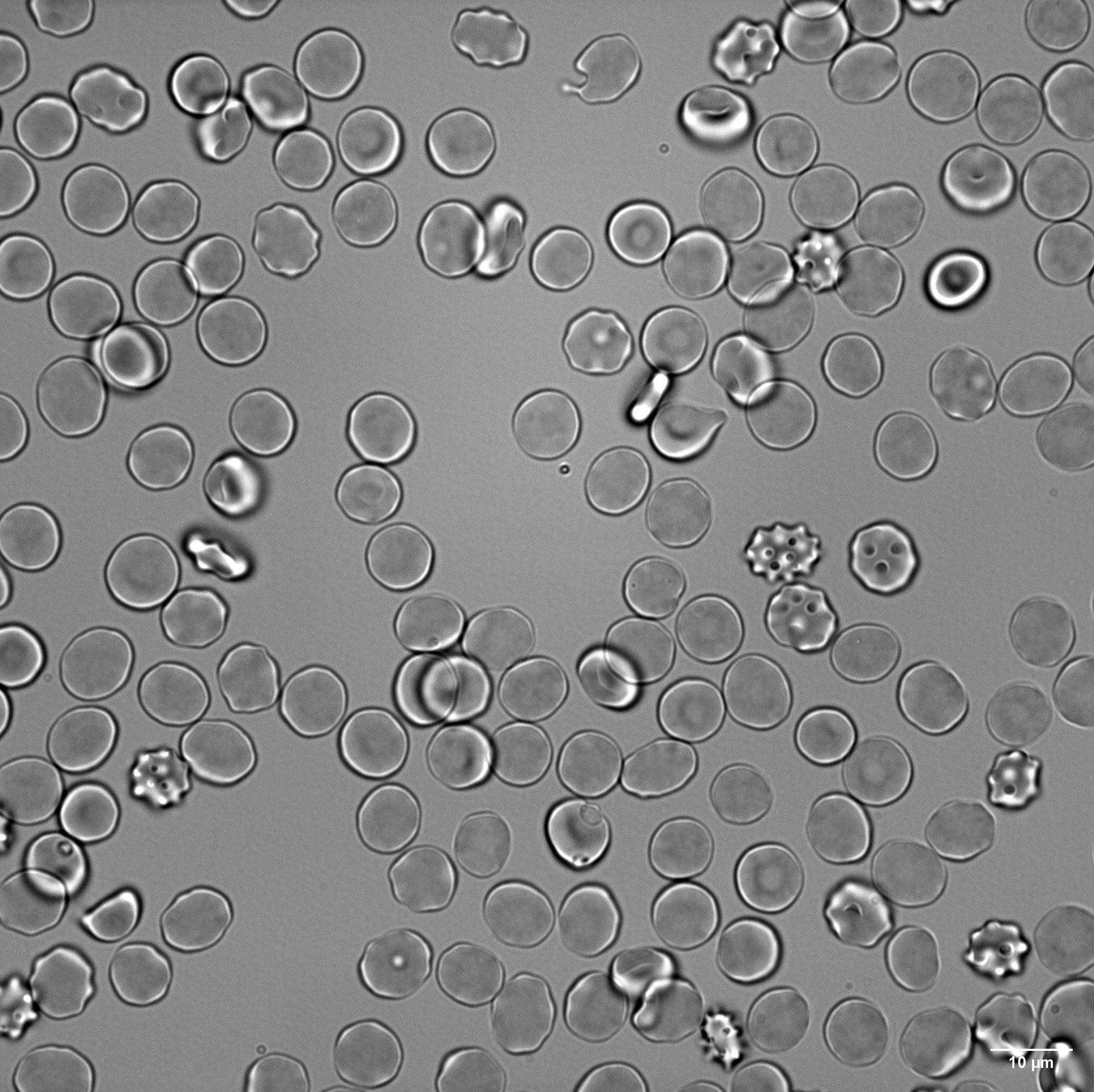

Supplement: Supplementary file 13 — Source data Fig. 2 [file 44319_2025_435_MOESM13_ESM.zip › 2+/2C +/live_Jask_2hpi.jpg]

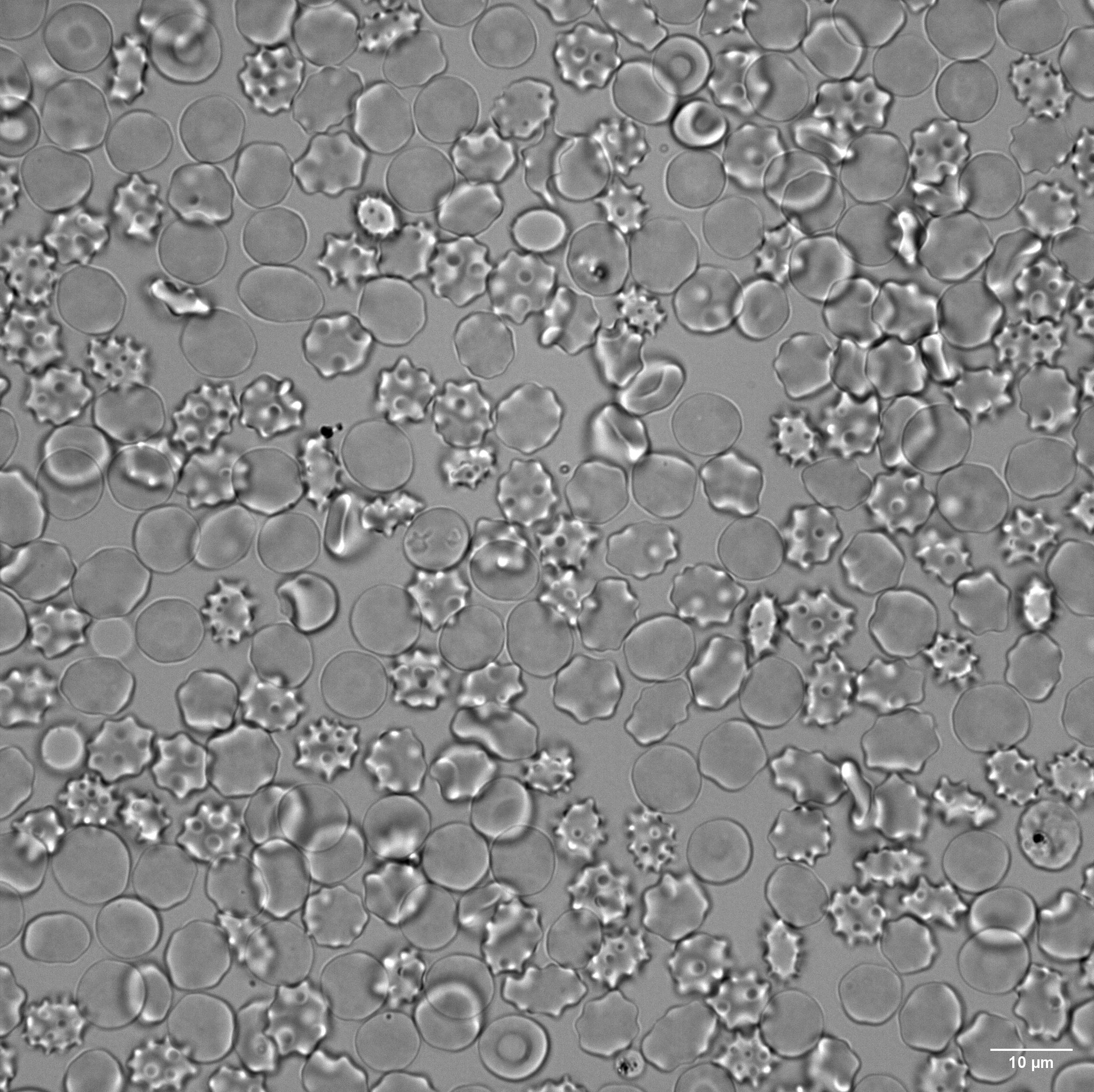

Supplement: Supplementary file 13 — Source data Fig. 2 [file 44319_2025_435_MOESM13_ESM.zip › 2+/2C +/Live_DMSO_2hpi.jpg]

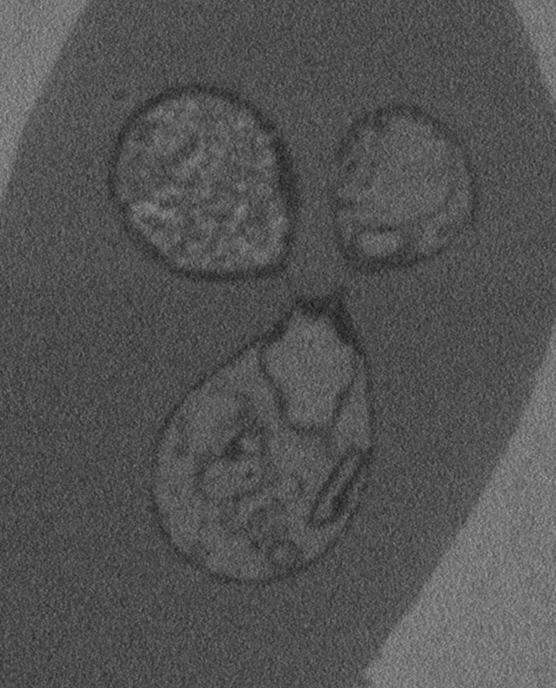

Supplement: Supplementary file 17 — Source data Fig. 4 [file 44319_2025_435_MOESM17_ESM.zip › 4+/4A+/EM3625_3_multiROI_set3_3VBSED_roi_00_slice_0036 crop.tif]

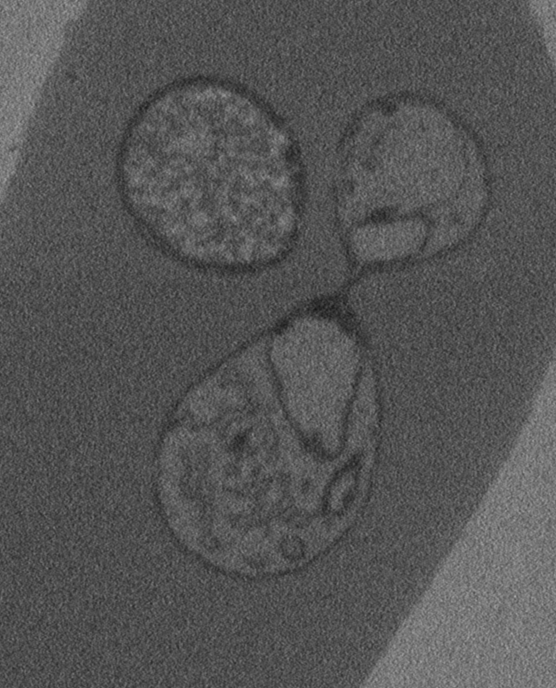

Supplement: Supplementary file 17 — Source data Fig. 4 [file 44319_2025_435_MOESM17_ESM.zip › 4+/4A+/EM3625_3_multiROI_set3_3VBSED_roi_00_slice_0037 crop.tif]

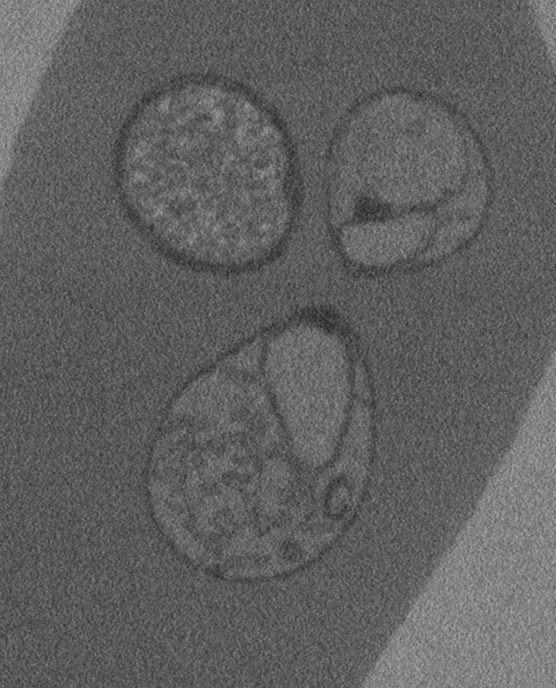

Supplement: Supplementary file 17 — Source data Fig. 4 [file 44319_2025_435_MOESM17_ESM.zip › 4+/4A+/EM3625_3_multiROI_set3_3VBSED_roi_00_slice_0038 crop.tif]

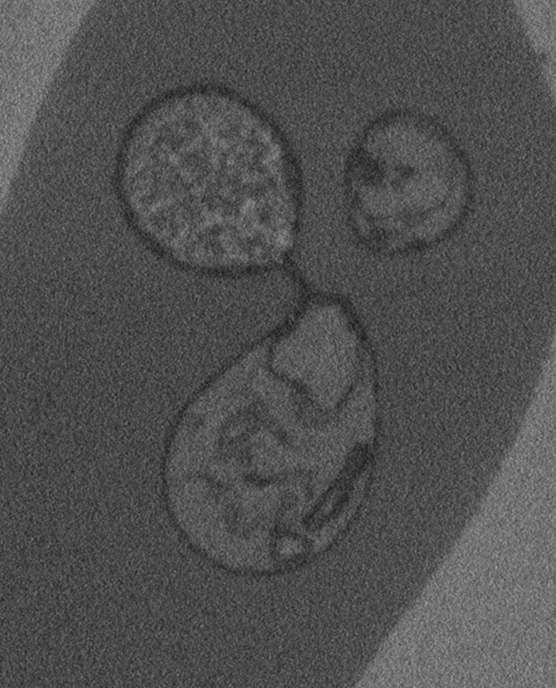

Supplement: Supplementary file 17 — Source data Fig. 4 [file 44319_2025_435_MOESM17_ESM.zip › 4+/4A+/EM3625_3_multiROI_set3_3VBSED_roi_00_slice_0035 crop.tif]

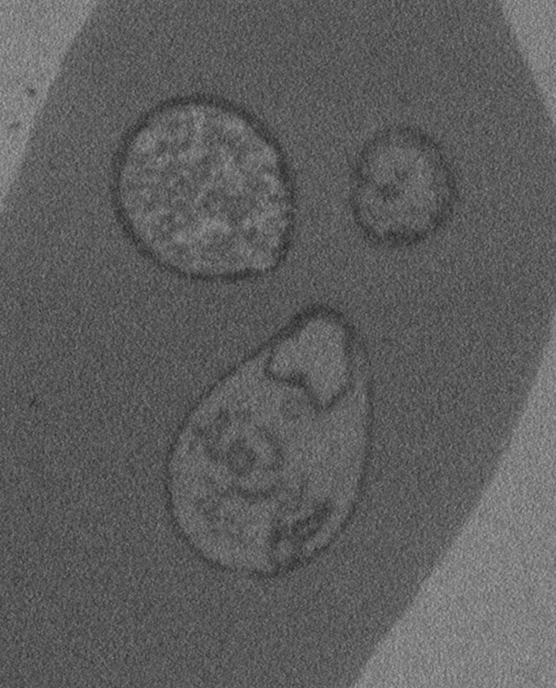

Supplement: Supplementary file 17 — Source data Fig. 4 [file 44319_2025_435_MOESM17_ESM.zip › 4+/4A+/EM3625_3_multiROI_set3_3VBSED_roi_00_slice_0034 crop.tif]

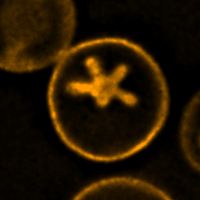

Supplement: Supplementary file 17 — Source data Fig. 4 [file 44319_2025_435_MOESM17_ESM.zip › 4+/4C+/717-008 slice 0016 orange RGB.tif]

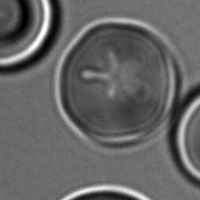

Supplement: Supplementary file 17 — Source data Fig. 4 [file 44319_2025_435_MOESM17_ESM.zip › 4+/4C+/717-008 slice 0016 white.tif]

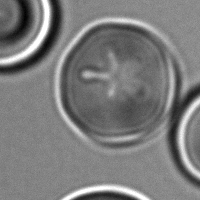

Supplement: Supplementary file 17 — Source data Fig. 4 [file 44319_2025_435_MOESM17_ESM.zip › 4+/4C+/717-008 slice 0016 whiteRGB.tif]

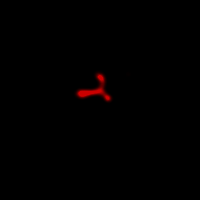

Supplement: Supplementary file 17 — Source data Fig. 4 [file 44319_2025_435_MOESM17_ESM.zip › 4+/4C+/717-008 slice 0016 redRGB.tif]

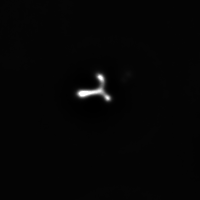

Supplement: Supplementary file 17 — Source data Fig. 4 [file 44319_2025_435_MOESM17_ESM.zip › 4+/4C+/717-008 slice 0016 red.tif]

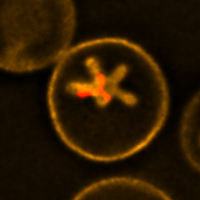

Supplement: Supplementary file 17 — Source data Fig. 4 [file 44319_2025_435_MOESM17_ESM.zip › 4+/4C+/717-008 slice 0016 orange red.tif (RGB).tif]

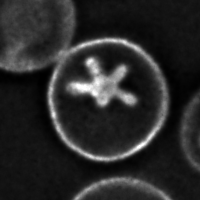

Supplement: Supplementary file 17 — Source data Fig. 4 [file 44319_2025_435_MOESM17_ESM.zip › 4+/4C+/717-008 slice 0016 orange.tif]

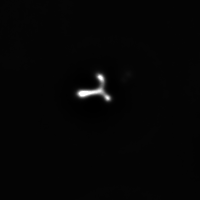

Supplement: Supplementary file 17 — Source data Fig. 4 [file 44319_2025_435_MOESM17_ESM.zip › 4+/4C+/717-008 slice 0016 orange red.tif]

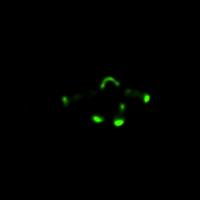

Supplement: Supplementary file 18 — Source data Fig. 5 [file 44319_2025_435_MOESM18_ESM.zip › 5+/5B+/717-17 Ginger slice 13 greenRGB.tif]

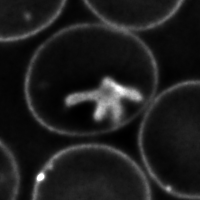

Supplement: Supplementary file 18 — Source data Fig. 5 [file 44319_2025_435_MOESM18_ESM.zip › 5+/5B+/717-17 Ginger slice 13 orange.tif]

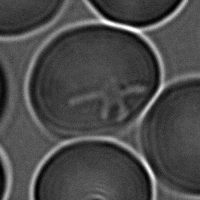

Supplement: Supplementary file 18 — Source data Fig. 5 [file 44319_2025_435_MOESM18_ESM.zip › 5+/5B+/717-17 Ginger slice 13 white.tif]

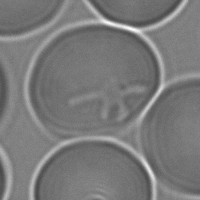

Supplement: Supplementary file 18 — Source data Fig. 5 [file 44319_2025_435_MOESM18_ESM.zip › 5+/5B+/717-17 Ginger slice 13 whiteRGB.tif]

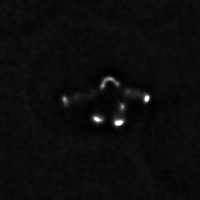

Supplement: Supplementary file 18 — Source data Fig. 5 [file 44319_2025_435_MOESM18_ESM.zip › 5+/5B+/717-17 Ginger slice 13 green.tif]

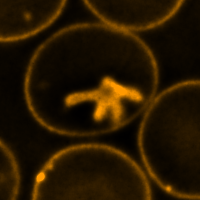

Supplement: Supplementary file 18 — Source data Fig. 5 [file 44319_2025_435_MOESM18_ESM.zip › 5+/5B+/717-17 Ginger slice 13 orangeRGB.tif]

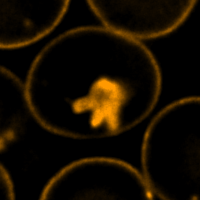

Supplement: Supplementary file 18 — Source data Fig. 5 [file 44319_2025_435_MOESM18_ESM.zip › 5+/5B+/717-018 slice 11 orangeRGB.tif]

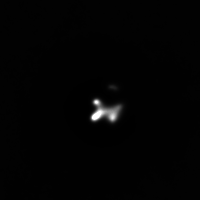

Supplement: Supplementary file 18 — Source data Fig. 5 [file 44319_2025_435_MOESM18_ESM.zip › 5+/5B+/717-018 slice 11 red orange green.tif]

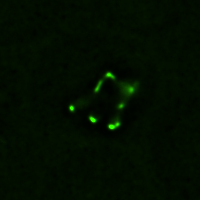

Supplement: Supplementary file 18 — Source data Fig. 5 [file 44319_2025_435_MOESM18_ESM.zip › 5+/5B+/717-018 slice 11 greenRGB.tif]

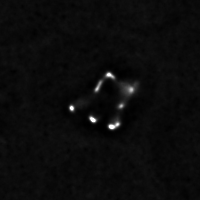

Supplement: Supplementary file 18 — Source data Fig. 5 [file 44319_2025_435_MOESM18_ESM.zip › 5+/5B+/717-018 slice 11 green.tif]

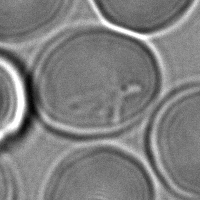

Supplement: Supplementary file 18 — Source data Fig. 5 [file 44319_2025_435_MOESM18_ESM.zip › 5+/5B+/717-018 slice 11 whiteRGB.tif]

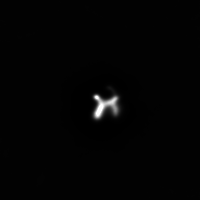

Supplement: Supplementary file 18 — Source data Fig. 5 [file 44319_2025_435_MOESM18_ESM.zip › 5+/5B+/717-17 Ginger slice 13 red orange green.tif]

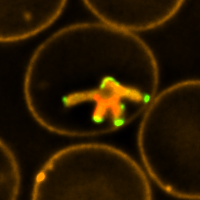

Supplement: Supplementary file 18 — Source data Fig. 5 [file 44319_2025_435_MOESM18_ESM.zip › 5+/5B+/717-17 Ginger slice 13 red orange green.tif (RGB).tif]

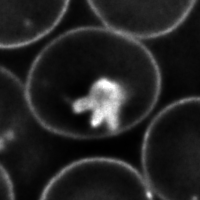

Supplement: Supplementary file 18 — Source data Fig. 5 [file 44319_2025_435_MOESM18_ESM.zip › 5+/5B+/717-018 slice 11 orange.tif]

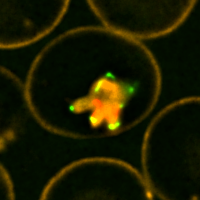

Supplement: Supplementary file 18 — Source data Fig. 5 [file 44319_2025_435_MOESM18_ESM.zip › 5+/5B+/717-018 slice 11 red orange greenRGB.tif (RGB) 320.tif]

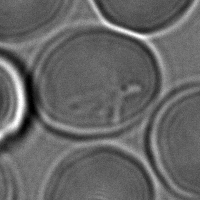

Supplement: Supplementary file 18 — Source data Fig. 5 [file 44319_2025_435_MOESM18_ESM.zip › 5+/5B+/717-018 slice 11 white.tif]

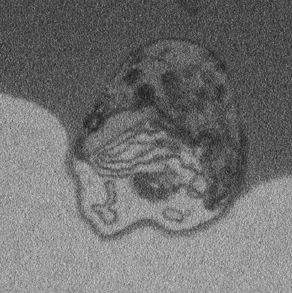

Supplement: Supplementary file 18 — Source data Fig. 5 [file 44319_2025_435_MOESM18_ESM.zip › 5+/5A+/EM section 2 small.tif]

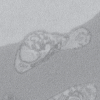

Supplement: Supplementary file 18 — Source data Fig. 5 [file 44319_2025_435_MOESM18_ESM.zip › 5+/5A+/DMSO 1 light.tif]

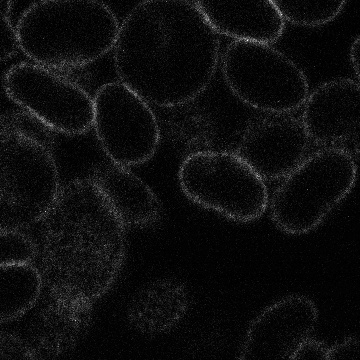

Supplement: Supplementary file 18 — Source data Fig. 5 [file 44319_2025_435_MOESM18_ESM.zip › 5+/5C +/R6_8/R6hpi_8_MERGE.tif]

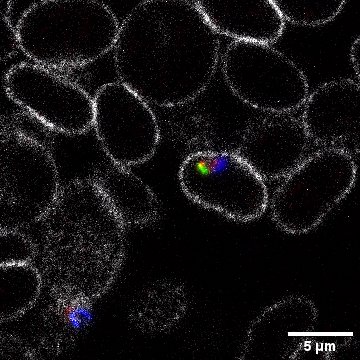

Supplement: Supplementary file 18 — Source data Fig. 5 [file 44319_2025_435_MOESM18_ESM.zip › 5+/5C +/R6_8/R6hpi_8_MERGE.jpg]

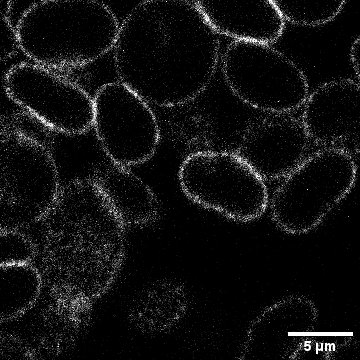

Supplement: Supplementary file 18 — Source data Fig. 5 [file 44319_2025_435_MOESM18_ESM.zip › 5+/5C +/R6_8/R6hpi_8_WGA_RBC.jpg]

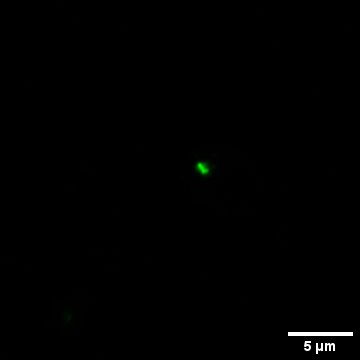

Supplement: Supplementary file 18 — Source data Fig. 5 [file 44319_2025_435_MOESM18_ESM.zip › 5+/5C +/R6_8/R6hpi_8_RESA.jpg]

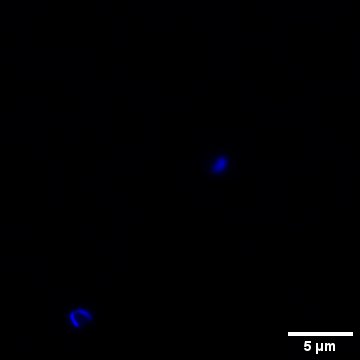

Supplement: Supplementary file 18 — Source data Fig. 5 [file 44319_2025_435_MOESM18_ESM.zip › 5+/5C +/R6_8/R6hpi_8_DAPI.jpg]

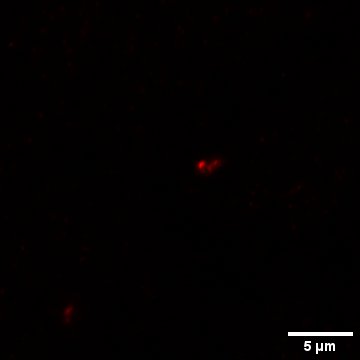

Supplement: Supplementary file 18 — Source data Fig. 5 [file 44319_2025_435_MOESM18_ESM.zip › 5+/5C +/R6_8/R6hpi_8_EXP2.jpg]

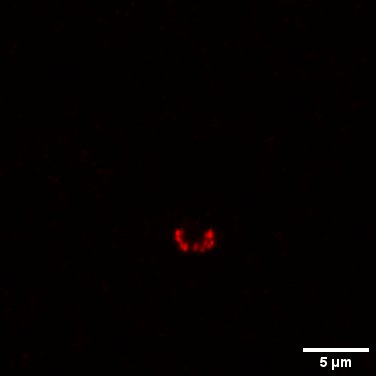

Supplement: Supplementary file 18 — Source data Fig. 5 [file 44319_2025_435_MOESM18_ESM.zip › 5+/5C +/D6_4/D6hpi_4_EXP2.jpg]

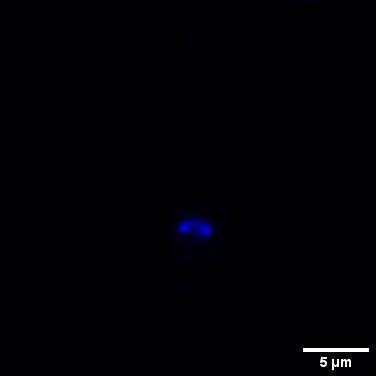

Supplement: Supplementary file 18 — Source data Fig. 5 [file 44319_2025_435_MOESM18_ESM.zip › 5+/5C +/D6_4/D6hpi_4_DAPI.jpg]

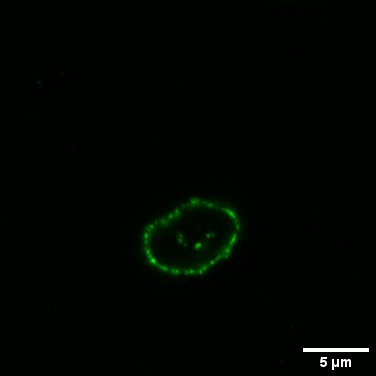

Supplement: Supplementary file 18 — Source data Fig. 5 [file 44319_2025_435_MOESM18_ESM.zip › 5+/5C +/D6_4/D6hpi_4_RESA.jpg]

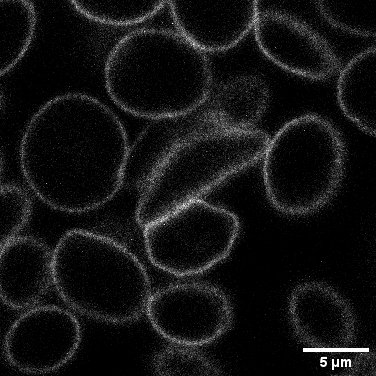

Supplement: Supplementary file 18 — Source data Fig. 5 [file 44319_2025_435_MOESM18_ESM.zip › 5+/5C +/D6_4/D6hpi_4_WGA_RBC.jpg]

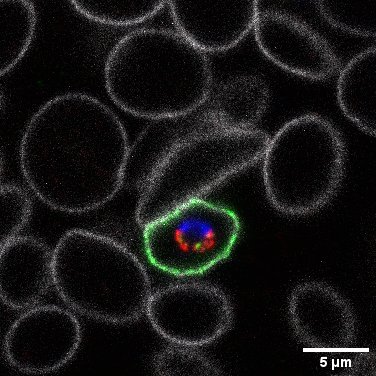

Supplement: Supplementary file 18 — Source data Fig. 5 [file 44319_2025_435_MOESM18_ESM.zip › 5+/5C +/D6_4/D6hpi_4_merge.jpg]
