# Supplementary material for: Malaria parasites undergo a rapid and extensive metamorphosis after invasion of the host erythrocyte
Source: EMBO Rep. 2025 Apr 4;26(10):2545–73. doi: 10.1038/s44319-025-00435-3 (PMC12116788; doi:10.1038/s44319-025-00435-3)
Supplement: Supplementary file 14 — Source data Fig. 3B-D [file 44319_2025_435_MOESM14_ESM.zip › 3B +/supp table EM models_18112023.docx]

| **# Model** | **Surface area (nm^2^)** | **Volume (µm^3^)** |
| --- | --- | --- |
| Merozoite (Schizont 1) | | |
| 1 | 9.3 | 2.3 |
| 2 | 9.0 | 2.1 |
| 3 | 8.6 | 2.0 |
| 4 | 9.5 | 2.3 |
| 5 | 8.7 | 2.2 |
| 6 | 8.7 | 1.9 |
| 7 | 9.2 | 2.3 |
| 8 | 8.1 | 2.0 |
| 9 | 6.2 | 1.5 |
| 10 | 8.2 | 1.9 |
| 11 | 7.8 | 1.8 |
| 12 | 10.0 | 2.1 |
| 13 | 9.2 | 2.2 |
| 14 | 9.2 | 2.1 |
| 15 | 9.0 | 2.2 |
| 16 | 8.3 | 1.9 |
| 17 | 8.9 | 2.0 |
| 18 | 9.7 | 2.1 |
| 19 | 9.8 | 2.1 |
| 20 | 9.0 | 2.1 |
| 21 | 8.8 | 2.0 |
| 22 | 8.2 | 1.9 |
| 23 | 8.3 | 1.9 |
| 24 | 9.2 | 2.2 |
| 25 | 8.5 | 2.0 |
| 26 | 9.2 | 2.0 |
| 27 | 8.9 | 2.0 |
| 28 | 8.2 | 1.9 |
| 29 | 8.8 | 2.1 |
| 30 | 2.1 | 1.7 |
| Merozoite (Schizont 2) | | |
| 1 | 8.8 | 2.2 |
| 2 | 8.6 | 2.2 |
| 3 | 8.8 | 2.2 |
| 4 | 9.0 | 2.1 |
| 5 | 8.5 | 2.0 |
| 6 | 9.6 | 2.4 |
| 7 | 10.1 | 2.3 |
| 8 | 9.3 | 2.2 |
| 9 | 9.2 | 2.2 |
| 10 | 9.6 | 2.2 |
| 11 | 9.2 | 2.0 |
| 12 | 2.1 | 2.3 |
| 13 | 9.8 | 2.4 |
| 14 | 9.0 | 2.1 |
| 15 | 8.7 | 2.1 |
| 16 | 9.6 | 2.4 |
| 17 | 9.3 | 2.2 |
| 18 | 8.8 | 2.1 |
| 19 | 8.9 | 2.1 |
| 20 | 9.2 | 2.2 |
| 21 | 9.4 | 2.3 |
| 22 | 8.7 | 2.0 |
| 23 | 8.6 | 2.0 |
| 24 | 9.1 | 2.1 |
| 25 | 9.1 | 2.1 |
| 26 | 8.7 | 2.1 |
| 27 | 9.4 | 2.3 |
| **Experiment 1:** |  |  |
| DMSO Rings 20 mins | | |
| 1 | 18.8 | 3.5 |
| 2 | 13.3 | 2.2 |
| 3 | 11.2 | 2.8 |
| 4 | 15.4 | 2.3 |
| 5 | 11.0 | 2.7 |
| 6 | 14.2 | 2.7 |
| 7 | 10.9 | 2.0 |
| 8 | 11.4 | 2.7 |
| 9 | 11.2 | 2.9 |
| 10 | 13.1 | 2.7 |
| 11 | 11.7 | 3.3 |
| 12 | 18.2 | 3.4 |
| 13 | 18.5 | 3.7 |
| 14 | 15.2 | 2.9 |
| 15 | 15.0 | 3.0 |
| 16 | 12.9 | 2.7 |
| RAPA Rings 20 mins | | |
| 1 | 16.2 | 3.9 |
| 2 | 13.9 | 3.2 |
| 3 | 9.8 | 2.5 |
| 4 | 15.9 | 3.2 |
| 5 | 16.5 | 3.7 |
| 6 | 11.6 | 3.1 |
| 7 | 9.9 | 2.6 |
| 8 | 10.2 | 2.5 |
| 9 | 9.9 | 2.4 |
| 10 | 15.7 | 3.8 |
| 11 | 9.7 | 2.5 |
| 12 | 10.8 | 3.0 |
| 13 | 8.7 | 2.1 |
| 14 | 15.3 | 3.7 |
| 15 | 9.6 | 2.4 |
| 16 | 16.2 | 3.6 |
| 17 | 10.1 | 2.7 |
| 18 | 9.4 | 2.4 |
| DMSO Rings 2 hours | |  |
| 1 | 16.8 | 3.3 |
| 2 | 20.3 | 3.1 |
| 3 | 21.5 | 3.0 |
| 4 | 16 | 2.3 |
| 5 | 17.8 | 3.5 |
| 6 | 20.1 | 3.6 |
| 7 | 16.5 | 2.5 |
| 8 | 22.4 | 3.7 |
| 9 | 18 | 3.8 |
| 10 | 13.6 | 3.3 |
| 11 | 20.9 | 4.3 |
| 12 | 16.5 | 4.0 |
| 13 | 14.8 | 4.4 |
| 14 | 18.6 | 3.9 |
| 15 | 18.8 | 3.3 |
| 16 | 14.5 | 3.1 |
| RAPA Rings 2 hours | |  |
| 1 | 12.7 | 3.9 |
| 2 | 14.2 | 3.8 |
| 3 | 14.9 | 4.1 |
| 4 | 14.7 | 4.4 |
| 5 | 9.0 | 2.3 |
| 6 | 13.2 | 3.8 |
| 7 | 25.8 | 4.3 |
| 8 | 12.8 | 3.8 |
| 9 | 15.2 | 5.1 |
| 10 | 15.3 | 5.1 |
| 11 | 14.2 | 4.3 |
| 12 | 11.8 | 3.5 |
| 13 | 16.3 | 5.3 |
| 14 | 14.9 | 4.9 |
| 15 | 13.1 | 3.5 |
| 16 | 13.2 | 4.3 |
| 17 | 12.6 | 3.7 |
| **Experiment 2:** |  |  |
| DMSO Rings |  |  |
| 1 | 28.90 | 5.32 |
| 2 | 23.17 | 5.15 |
| 3 | 19.00 | 2.66 |
| 4 | 14.80 | 2.55 |
| 5 | 14.00 | 2.31 |
| 6 | 12.10 | 1.38 |
| 7 | 25.80 | 4.75 |
| 8 | 19.60 | 3.03 |
| 9 | 15.40 | 2.75 |
| RAPA Rings |  |  |
| 1 | 10.10 | 2.71 |
| 2 | 8.49 | 2.11 |
| 3 | 11.00 | 2.78 |
| 4 | 8.21 | 1.59 |
| 5 | 10.50 | 2.39 |
| 6 | 7.57 | 1.69 |
| 7 | 11.40 | 2.58 |
