# Supplementary material for: Malaria parasites undergo a rapid and extensive metamorphosis after invasion of the host erythrocyte
Source: EMBO Rep. 2025 Apr 4;26(10):2545–73. doi: 10.1038/s44319-025-00435-3 (PMC12116788; doi:10.1038/s44319-025-00435-3)
Supplement: Supplementary file 20 — Expanded View Figures [file 44319_2025_435_MOESM20_ESM.pdf]

Expanded View Figures

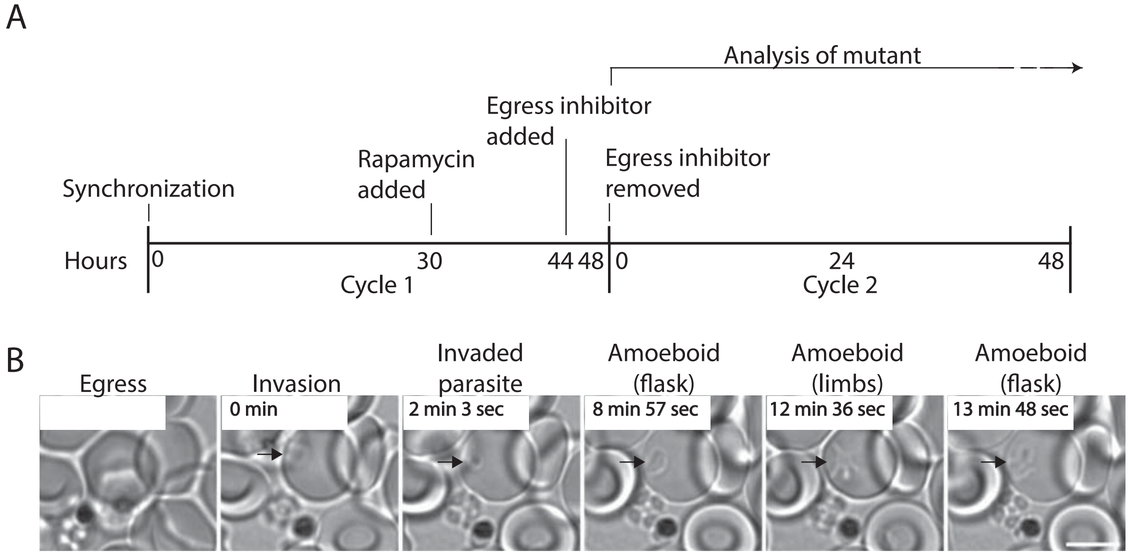

Figure EV1. Phenotypic analyses of parasites lacking PV6.

(**A**) Schematic representation of the protocol used to analyse the phenotype of the *P. falciparum* PV6 DiCre parasites. Synchronized parasites were treated with a minimum of 10 nM rapamycin (or an equivalent volume of DMSO) for 1 h at the trophozoite stage (~30 h post-invasion) in the first cycle. Close to the end of the first cycle, an egress inhibitor (either Compound 2 or ML10) was added to arrest the parasites at a very late schizont stage. When most of the parasites appeared arrested, the egress inhibitor was removed to initiate a round of synchronized invasion, starting cycle 2, allowing for observation of the phenotype of PV6 over time. (**B**) Live-cell imaging of amoeboid formation. The frame rate was set at one frame/second, time starting from invasion is indicated in each panel. The arrows indicate the parasite. Scale bar represents 5 µm.

© The Author(s)

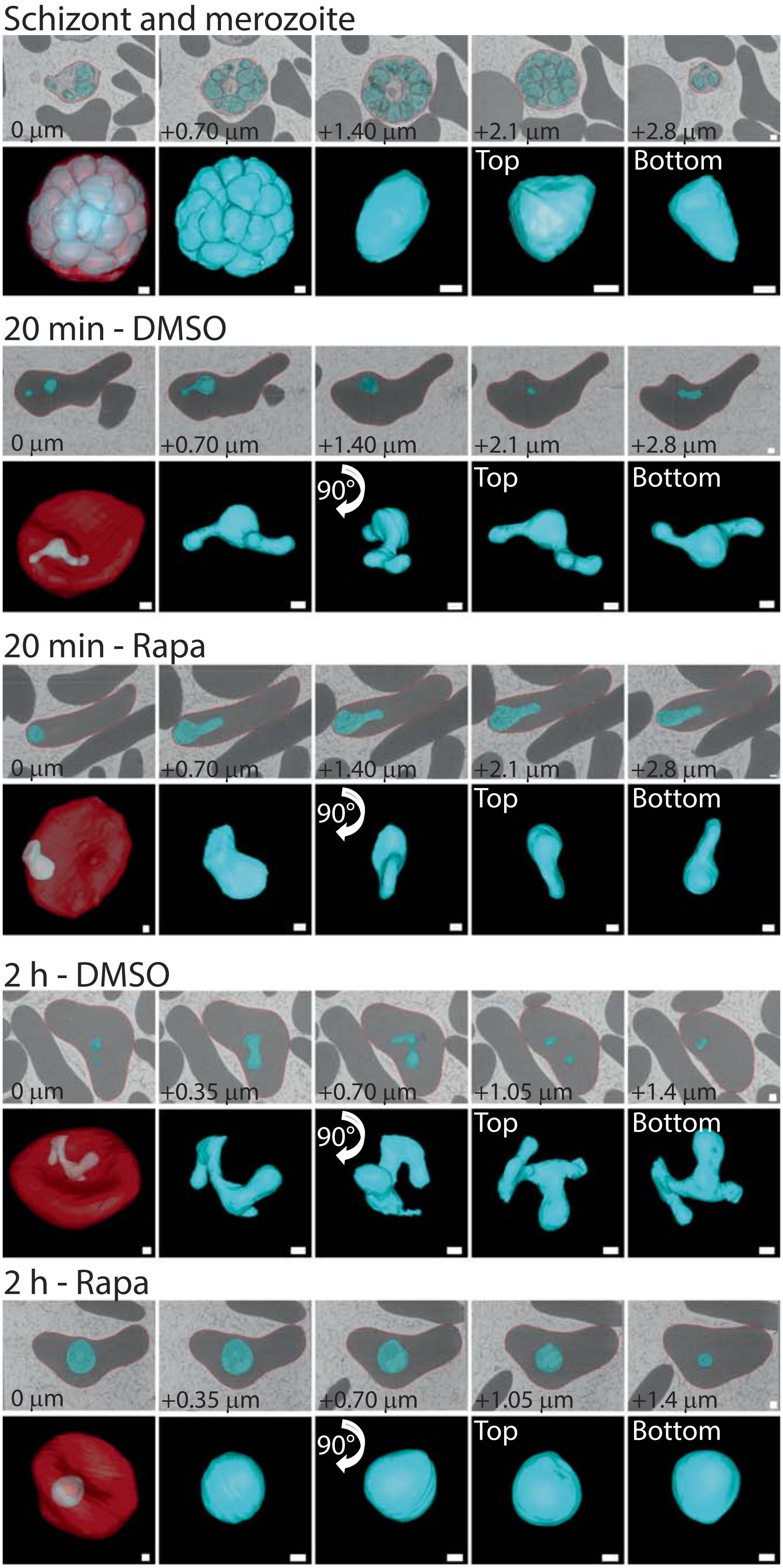
Schizont and merozoite
0 µm
+0.70 µm
+1.40 µm
+2.1 µm
+2.8 µm
Top
Bottom
20 min - DMSO
0 µm
+0.70 µm
+1.40 µm
+2.1 µm
+2.8 µm
90°
Top
Bottom
20 min - Rapa
0 µm
+0.70 µm
+1.40 µm
+2.1 µm
+2.8 µm
90°
Top
Bottom
2 h - DMSO
0 µm
+0.35 µm
+0.70 µm
+1.05 µm
+1.4 µm
90°
Top
Bottom
2 h - Rapa
0 µm
+0.35 µm
+0.70 µm
+1.05 µm
+1.4 µm
90°
Top
Bottom

 © The Author(s)

Figure EV2. Additional three-dimensional views of the models presented in Fig. 3.

Three-dimensional models from SBF-SEM data of the parasites presented in Fig. 3, illustrating the shape of the parasite (cyan) and its positioning within the erythrocyte (red) and additional SEM-SBF sections. The scale bars represent 500 nm.

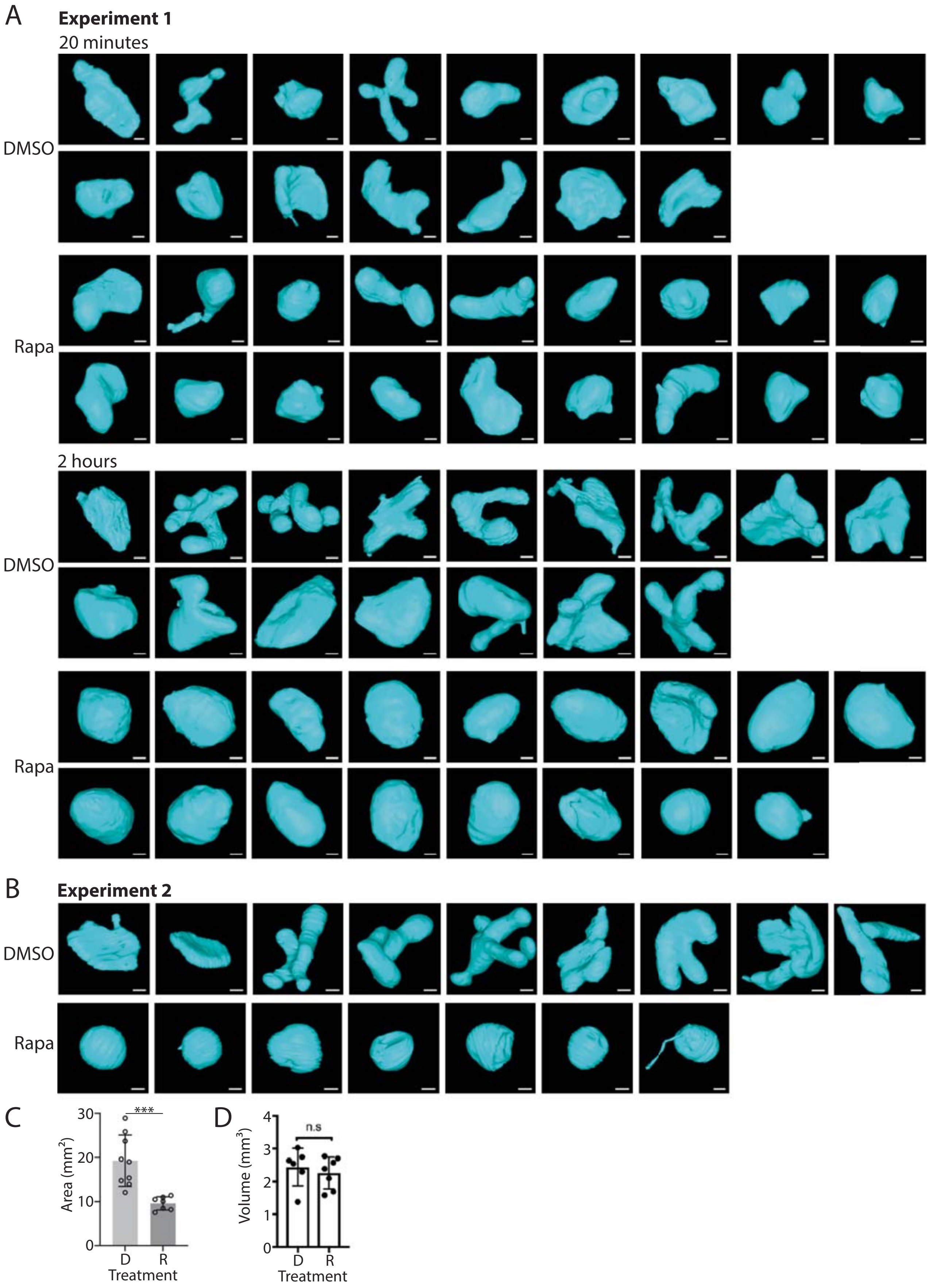
A
Experiment 1
20 minutes
DMSO
Rapa
2 hours
DMSO
Rapa
B
Experiment 2
DMSO
Rapa
C
Area (mm²)
***
30
20
10
0
D
R
Treatment
D
Volume (mm³)
n.s
4
3
2
1
0
D
R
Treatment

Figure EV3. Three-dimensional models of PfBLD529 parasites treated with DMSO or rapamycin.

(**A**) These models were obtained in the same experiment as those presented in Fig. 3 and Fig. EV2. The invasion of these parasites was carefully synchronized using an egress inhibitor to allow the development of the parasites to be followed over time. Time indicated refers to the time after removal of egress inhibitor. The scale bars represent 500 nm. (**B**) Three-dimensional models from SBF-SEM data of DMSO-treated and rapamycin-treated parasites for which invasion was not synchronized. The parasites had been synchronized at the start of cycle 1 (Fig. EV1) and were allowed to progress to the next cycle without further synchronization. The scale bars represent 500 nm. (**C**) Surface area of the parasites shown in panel B; D-DMSO, R-rapamycin. Error bars +/− SD. Data represent the measurement of at least 7 parasites. The Mann–Whitney U test was performed for statistical analysis (***$P < 0.001$, $P = 0.0002$). (**D**) Volume of the parasites shown in (**B**). Error bars +/− SD. Data represent the measurement of at least 7 parasites. The Mann–Whitney U test was performed for statistical analysis. No significance difference was detected between the wild-type parasites and the parasites lacking PV6.

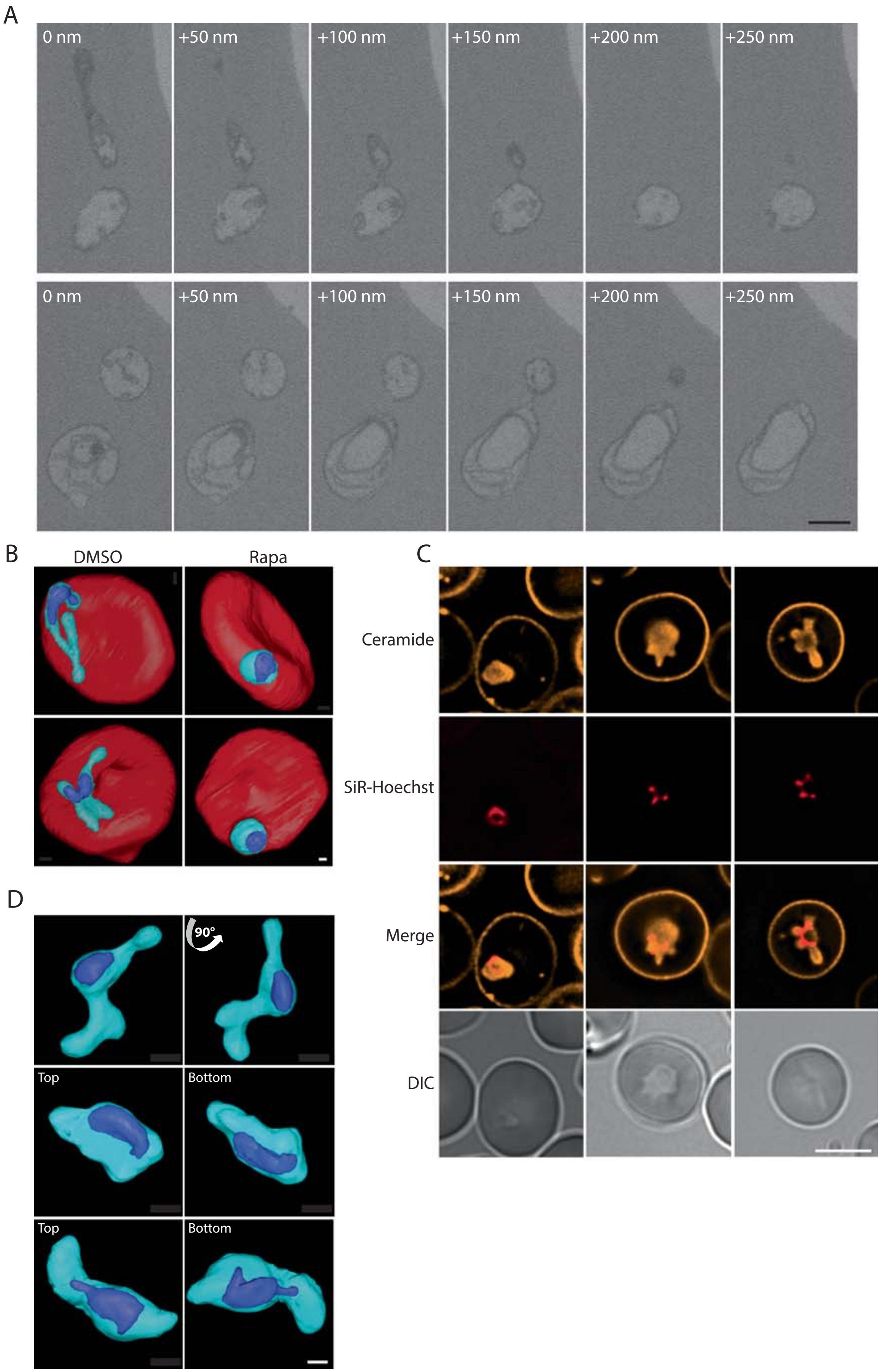
A
0 nm
+50 nm
+100 nm
+150 nm
+200 nm
+250 nm
B
DMSO
Rapa
C
Ceramide
SiR-Hoechst
Merge
DIC
D
90°
Top
Bottom
Top
Bottom

◀ **Figure EV4. Nuclear morphology in wild-type amoeboid-shaped parasites and parasites lacking PV6.**

(**A**) Consecutive SBF-SEM sections, showing the thin connections between limbs of two different parasites. Numbers in the upper left-hand corner of the panels indicate distance between the sections and the scale bar represents 500 nm. (**B**) Three-dimensional models from SBF-SEM data of erythrocytes infected with DMSO-treated and rapamycin-treated parasites 2 h after removal of egress inhibitor. Erythrocytes (red), parasite (cyan) and the nucleus (blue) are highlighted. The scale bar represents 500 nm. (**C**) Live-cell fluorescence imaging of infected erythrocytes labelled with C5-Bodipy-ceramide (orange) and SiR-Hoechst (red) 2 h after removal of egress inhibitor. Note that in the merged imaged, the parasite and the SiR-Hoechst do not overlap perfectly owing to the movement of the parasite during the acquisition of the images. The scale bar represents 5 µm. (**D**) Three-dimensional model showing the nucleus (blue) in wild-type parasites (magenta) 20 min after removal of egress inhibitor. Scale bar represents 500 nm.

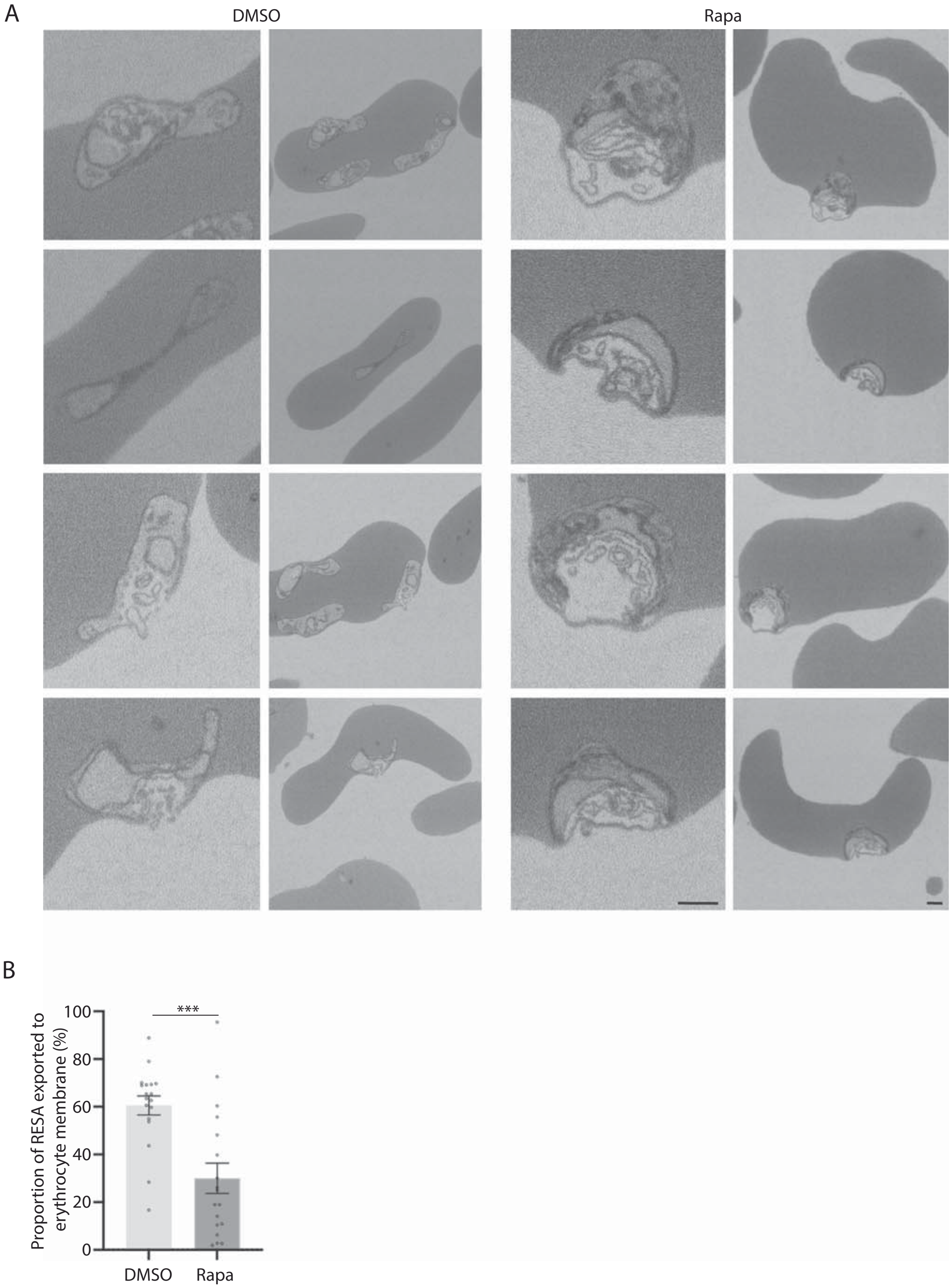
A
DMSO
Rapa
B
Proportion of RESA exported to erythrocyte membrane (%)
***
100
80
60
40
20
0
DMSO
Rapa

 © The Author(s)

Figure EV5. Additional SBF-SEM images of wild-type parasites and parasites lacking PV6.

(**A**) Individual SBF-SEM sections of different erythrocytes infected with wild-type PfBLD529 parasites (left; DMSO) and PfBLD529 parasites lacking PV6 (right; Rapa). In each case, the left-hand panel shows a close view of the parasite and the right-hand panel shows the entire infected erythrocyte. Note the accumulation of membranous whorls next to the parasites lacking PV6. The close views of the parasites in the top row are also shown in Fig. 5. Scale bars represent 500 nm. (**B**) Analysis of export of RESA in erythrocytes infected with DMSO-treated and rapamycin-treated parasites. Samples prepared for IFA were imaged as described in the Methods section. The amount of RESA exported to the erythrocyte was measured for 18 parasites obtained from two independent experiments. Error bars +/− SD. The Mann–Whitney U test was performed for statistical analysis (***$P < 0.001$ ($P = 0.0005$)).

© The Author(s)

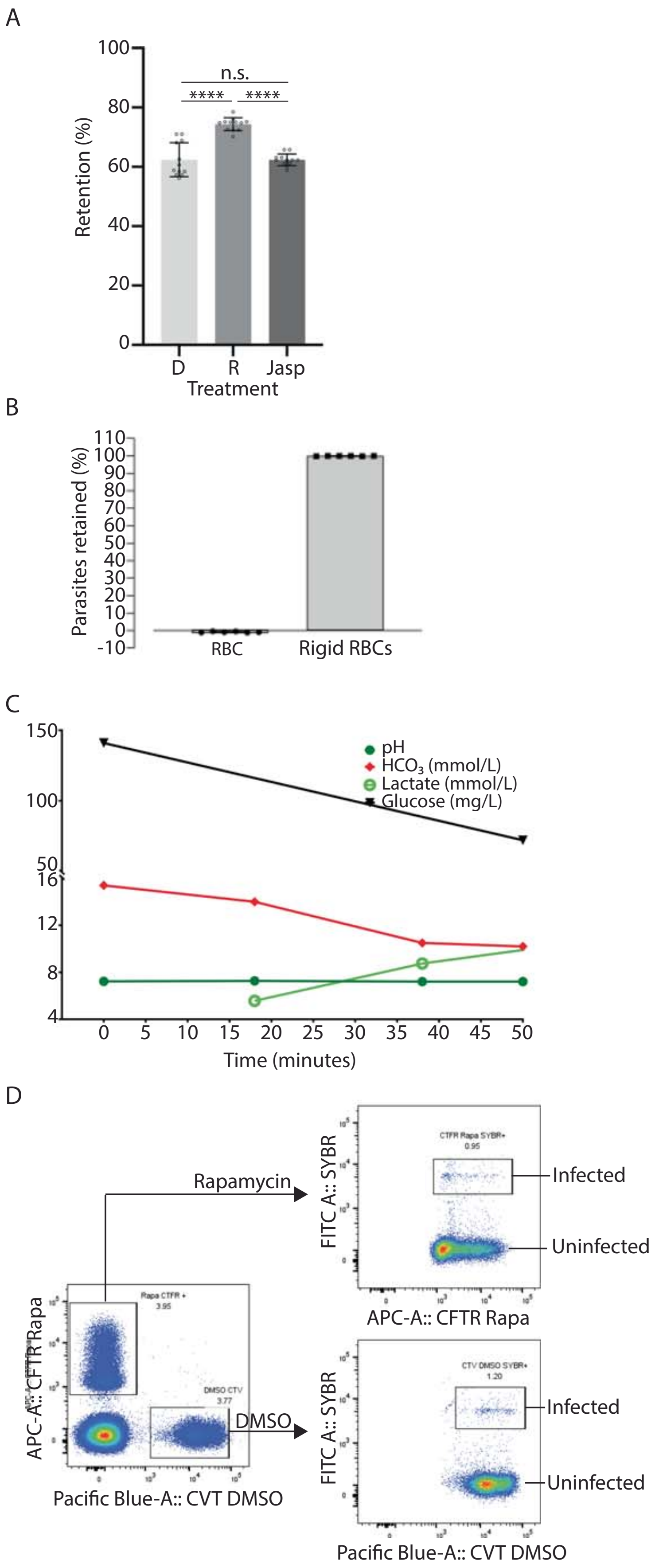
A
Retention (%)
n.s.
****
****
D
R
Jasp
Treatment
B
Parasites retained (%)
RBC
Rigid RBCs
C
pH
HCO3 (mmol/L)
Lactate (mmol/L)
Glucose (mg/L)
Time (minutes)
D
Rapamycin
DMSO
FITC A:: SYBR
Infected
Uninfected
APC-A:: CFTR Rapa
Pacific Blue-A:: CVT DMSO

© The Author(s)

Figure EV6. Analysis of samples used for microsphiltration and ex vivo spleen perfusion.

(**A**) Retention of PV6-diCre parasites cultures treated with DMSO, rapamycin or jasplakinolide. Data represent 2 biological replicates with a minimum of 6 technical replicates. Error bars: +/− SD. The Mann–Whitney U test was performed for statistical analysis: ns, not significant; $****P < 0.0001$. (**B**) Retention of untreated erythrocytes (RBCs) and fixed erythrocytes (rigid RBCs) on the columns used for the microsphiltation analysis of erythrocyte infected with wild-type parasites or parasites lacking PV6. Data represents 1 biological replicate with 6 technical replicates. (**C**) Physiological state of ex vivo spleen during the course of the perfusion with infected erythrocytes. The pH and levels of bicarbonate, lactate and glucose were measured periodically to ensure the proper functioning and viability of the spleen. (**D**) Gating strategy used for cytometric analysis of blood passaged through ex vivo spleen.
